# Supplementary material for: Therapeutic targeting of measles virus polymerase with ERDRP-0519 suppresses all RNA synthesis activity
Source: PLoS Pathog. 2021 Feb 23;17(2):e1009371. doi: 10.1371/journal.ppat.1009371 (PMC7935272; doi:10.1371/journal.ppat.1009371)
Supplement: S1 Fig — MeV (NP_056924.1), HPIV3 (ARA15380.1), HPIV1 (ARB07783.1), NiV (AAY43917.1), MuV (AWI67642.1), PIV5 (YP_138518.1), RSV (YP_009518860.1), HMPV (Q6WB93.1), VSV (Q98776.1), and EBOV (NP_066251.1) L proteins were aligned using Clustal-Omega [66]. Positions of the RdRP (cyan), capping (green), connector (yellow), MTase (orange), and C-terminal (pink) domains are shown as colored bars above the sequences. Positions of ERDRP-0519 and GHP-88309 resistance mutations are highlighted in pink and salmon, respectively. Locations of the proposed RdRP and PRNTase motifs are labeled highlighted in purple and blue, respectively. The residues surrounding the binding pocket of ERDRP-0519 are boxed out (black lines). (PDF) [file ppat.1009371.s001.pdf]

```
MeV      -----------MDS-----LSVNQ----------ILYPEVHLDSPIVTNKIVAILEYA-RVPHAYSLEDPTLCQNIKHRLKNGFSNQMIINNVEVGNVIKSKLRSYPAHSH------IPY 87
HPIV3    -MDTE------SNN-----GTVSD----------ILYPECHLNSPIVKGKIAQLHTIM-SLPQPYDMDDDSILVITRQKIKLNKLDKRQRSIRRLKLILTEKVSDLGKYTF------IRY 91
HPIV1    -MDKL------EST-----QNSSD----------ILYPECHLNSPIVKSKIAQLHVLL-DINQPYDLKDNSIINITKYKIRNGGLSPRQIKIRSLGKILKQEIKDIDRYTF------EPY 91
Nipah    ----M------ADE-----LSISD----------IIYPECHLDSPIVSGKLISAIEYA-QLKHNQPSDDKRLSENIRLNLHGKRKSLYILRQSKQGNYIRNNVKNLKEFMH------IAY 88
Mumps    -------------M-----AGLNE----------ILLPEVHLNSPIVRYKLFYYILHG-QLPNDLEPDDLGPLANQNWKAIRAEESQVHARLKQIRVELIARIPSLRWTRSQREIAILIW 91
PIV5     -------------M-----AGSRE----------ILLPEVHLNSPIVKHKLYYYILLG-NLPNEIDLDDLGPLHNQNWNQIAHEESNLAQRLVNVRNFLITHIPDLRKGHWQEYVNVILW 91
RSV      -MDPI------ING------NSAN----------VYLTDSYLKGVISFSECNAL-GSY-IFNGPYLKNDYTNLISRQNPLIE------HMNLKK-LNITQSLISKYHKGEIK-----LEE 83
HMPV     -MDP-------LNE------STVN----------VYLPDSYLKGVISFSETNAI-GSC-LLKRPYLKNDNTAKVAIENPVIE------HVRLKN-AVNSKMKISDY-----K-----VVE 77
VSV      -MEVHDFETDEFNDFNEDDYATREFLNPDERMTYLNHADYNLNSPLISDDIDNLIRKFNSLPIPSMWD-S-----KNWDGVL-------------------------------------- 75
RABV     MLDPGEVYDDPIDPIELE----AEPRGTPIVPNILRNSDYNLNSPLIEDPARLMLEWLKTGNRPYRMTLT-DNCSRSFRVLK-------------------------------------- 77
EBOV     -------------------MATQH----------TQYPDARLSSPIVLDQCDLVTRAC-GLYSSYSLNPQ---------LRNCKLPKHIYRLK-YDVTVTKFLSDVPVA-------TLPI 73
                                .              :  *.. :

MeV      PNC-NQDLF-NI---EDKESTRK-------IRELLKKGNSLYSKVSDKV--FQ-----------------------------CLRDTNSRLGLGS------EL----REDIKEKIINL-- 152
HPIV3    PEM-SKEMF-KL---HIPGINSK-------VTELLLKADRTYSQMTDGL--RD-----------------------------LWINVLSKLASKN------DGSNYDLNEEINNISKV-- 160
HPIV1    PIF-SLELL-RL---DIPEICDK-------IRSIFSVSDRLIRELSSGF--QE-----------------------------LWLNILRQLGCVE------GKEGFDSLKDVDIISDI-- 160
Nipah    PEC-NNTLF-SI---TSQGMTSK-------LDNIMRKSFKAYNIISKKV--IG-----------------------------MLQNITRNLITQD------RRDEIINIHECRRLGDL-- 157
Mumps    PRI-LPILQ-AY---DLRQSMQL-------PTVWEKLTQSTVNLISDGL--ER-----------------------------VVLHISNQLTGKPNLFTRSRAGQGANDCSIPSTREL-- 166
PIV5     PRI-LPLIP-DF---KINDQLPL-------LKNWDKLVKESCSVINAGT--SQ-----------------------------CIQNLSYGLTGRGNLFTRSRELSGDRRD--IDLKTV-- 164
RSV      PTYFQSLLMTYKSMTSSEQIATT-NLLKKIIRRAIEISDVKVYAILNKLGLKEKDKIKSNNGQDEDNSVITTIIKDDI--LSAVKDNQSHLKADKNHS--------TKQKDTIKTTLLKK 192
HMPV     PVNMQHEIMKNV------HSCEL-TLLKQFLTRSKNISTLKLNMICDWLQLKST-----------------------------------------------------SD-D---TSI--L 131
VSV      ------EMLTSC------QANP-------------ISTSQMHKWMGSWLMSDN---HDASQG----YSFLHEVDKEAEITFDVVETFIRGWGNKP------------------------- 138
RABV     ------DYFKKV------DLGSL-----------KVGGMAAQSMISLWLYGAH---SESNRS----RRCITDLAHFYSKSSPIEKLLNLTLGNRGLRI--------------P-PEGV-- 150
EBOV     DFI-VPVLLKAL---SGNGFCPVEPRCQQFLDEIIKYTM------QDALFLKY-----------------------------YLKNVGAQEDCVDEHF----------------QEKI-- 136

MeV      --GVYMHSSQWFEPF-----------------------LFWFTVKTEMRSVIKSQTH---TCHRRR-HTPVFFTGSSVELLISRDLV------------------AIIS----KESQHVY 221
HPIV3    --HTAYKSDKWYNPF-----------------------KTWFTIKYDMRRLQKARNE---VTFNMG-KDYNLLEDQKNLLLIHPELV------------------LILD----KQNYNGY 229
HPIV1    --TDKYNKNTWYRPF-----------------------LTWFSIKYDMRWMQKNKSG---NHLDVS-NSHNFLDCKSYILIIYRDLV------------------IIIN----KLKLTGY 229
Nipah    --GKNMSQSKWYECF-----------------------LFWFTIKTEMRAVIKNSQK---PKFRSD-SCIIHMRDKSTEIILNPNLI------------------CIFKSD--KTGKKCY 228
Mumps    --SQIWFNNEWSGPV-----------------------KTWLMIKYRMRQLITNQKT---G--ELT-DLVTIVDTRSTLCIITPELV------------------ALYS----NEHKALT 233
PIV5     --VAAWHDSDWKRIS-----------------------DFWIMIKFQMRQLIVRQTD---H--NDS-DLITYIENREGIIIITPELV------------------ALFN----TENHTLT 231
RSV      LMCSMQHPPSWL--------------------------IHWFNLYTKLNNILTQYRSNE-V-KNHGFTLIDNQTLSGFQFI------------------LNQYGCIVYH----KELKRIT 262
HMPV     SFIDVEFIPSWV--------------------------SNWFSNWYNLNKLILEFRREE-V-IRTGSILC--RSLGKLVFI------------------VSSYGCIVKS----NKSKRVS 199
VSV      --IEYIKKERWTDSFK-ILAYLCQKFLDLHKLTLILNAVSEVELLNLARTFKGKVRR-----SSHGTNICRIRVPSLGPTFISEGWA--------------------------YFKKLDI 224
RABV     --LSCLERVDYDNAFGRYLANTYSSYLFFHVITLYMNALDWDEEKTILALW-KDLTS-----VDIGKDLVKFKDQIWGLLIVTKDFV--------------------------YSQSSNC 236
EBOV     --LSSIQGNEFLHQM-----------------------FFWYDLAILTRRGRLNRGNSRSTWFVHD-DLIDILGYGDYVFWKIPISMLPLNTQGIPHAAMDWYQASVFKEAVQGHTHIVS 230
                   :

MeV      YLTFELVLMYCDVIEGRLMTET--AMTIDARYAEL-----------LGRVRYMWKLIDGFFPALGNPTYQIVAMLEPLSLAYL-QLRDITVEL---RGAFLNHCFTEIHDVLDQNG---F 321
HPIV3    LITPELVLMYCDVIEGRWNISA--CAKLDPKLQSM-----------YQKGNNLWEVIDKLFPIMGEKTFDVISLLEPLALSLI-QTHDPVKQL---RGAFLNHVLSEMELIFESRE---S 329
HPIV1    VLTPELVLMYCDVVEGRWNMSS--AGRLDKRSSKI-----------TYKGEELWELIDSLFPNLGEDVYNIISLLEPLSLALI-QLDDPVTNL---KGAFMRHVLTELHTILIKDN---I 329
Nipah    YLTPEMVLMYCDVLEGRMMMET--TIKSDIKYQPL-----------ISRSNALWGLIDPLFPVMGNRIYNIVSMIEPLVLALL-QLKDEARIL---RGAFLHHCIKEMHQELSECG---F 328
Mumps    YLTFEMVLMVTDMLEGRLNVSS--LCTASHYLSPL-----------KKRIEILLTLVDDLALLMGDKVYGVVSSLESFVYAQL-QYGDPVIDI---KGTFYGFICNEILDLLTEDN---I 333
PIV5     YMTFEIVLMVSDMYEGRHNILS--LCTVSTYLNPL-----------KKRITYLLSLVDNLAFQIGDAVYNIIALLESFVYAQL-QMSDPIPEL---RGQFHAFVCSEILDALRGTN---S 331
RSV      VTTYNQFLTWKDISLSRLNVCL--ITWISNCLNTLNKSLGLRCGFNNVILTQLFLYGDCILKLFHNEGFYIIKEVEGFIMSLI-LNITEEDQF---RKRFYNSMLNNITDAANKAQKNLL 376
HMPV     FFTYNQLLTWKDVMLSRFNANF--CIWVSNSNLNENQEGLGLRSNLQGMLTNKLYETVDYMLSLCCNEGFSLVKEFEGFIMSEI-LRITEHAQF---STRFRNTLLNGLTDQLTKLKNKNR 313
VSV      LMDRNFLLMVKDVIIGRMQTVLSMVCRIDNLFSEQ----------DIFSLLNIYRIGDKIVERQGNFSYDLIKMVEPICNLKLMKLARESRPLVPQFPHFENHIKTSVDEGAK-ID---- 329
RABV     LFDRNYTLMLKDLFLSRFNSLMVLLSPPEPRYSDD----------LISQLCQLYIAGDQVLSMCGNSGYEVIKILEPYVVNSLVQRAEKFRPLIHSLGDFPVFIKDKVSQLEETFG---- 342
EBOV     VSTADVLIMCKDLITCRFNTTL--ISKIAEIEDPVCSDYP-----NFKIVSMLYQSGDYLLSILGSDGYKIIKFLEPLCLAKI-QLCSKYTER---KGRFLTQMHLAVNHTLEEITEMRA 339
             :  :   *:   *                                   :    * .     .  : ::  .*      :                *       :

MeV      SDE------------G---T---YHELIEALDYIFITDDIHLTGEIFSFFRSFGHPRLEAVTAAENVRKYMNQPKVIVYETLMKGHAIFCGII-INGYRDRHGGSWPPLTLPLHAADTIR 422
HPIV3    IKE------------F---L---SVDYIDKILDIFDKSTIDEIAEIFSFFRTFGHPPLEASIAAEKVRKYMYIEKQLKFDTINKCHAIFCTII-INGYRERHGGQWPPVTLPDHAHEFII 430
HPIV1    YTD------------S---E---ADSIMESLIKIFRETSIDEKAEIFSFFRTFGHPSLEAITAADKVRTHMYSSKKIILKTLYECHAIFCTII-INGYRERHGGQWPPCEFPNHVCLELK 430
Nipah    TDQ------------K---I---RSMFIDDLLSILNIDNIHLLAEFFSFFRTFGHPILEAKVAAEKVREHMLADKVLEYAPIMKAHAIFCGTI-INGYRDRHGGAWPPLYLPAHASKHII 429
Mumps    FTE------------E---E---ANKVLLDLTSQFDNLSPDLTAELLCIMRLWGHPTLTASQAASKVRESMCAPKVLDFQTIMKTLAFFHAIL-INGYRRSHNGIWPPTTLHGNAPKSLI 434
PIV5     FTQ------------D---E---LRTVTTNLISPFQDLTPDLTAELLCIMRLWGHPMLTASQAAGKVRESMCAGKVLDFPTIMKTLAFFHTIL-INGYRRKHHGVWPPLNLPGNASKGLT 432
RSV      SRVCHTLLDKTVSDNIINGRWIIILLSKFLKLIKLAGDNNLNNLSELYFLFRIFGHPMVDERQAMDAVKINCNETKFYLLSSLSMLRGAFIYRI-IKGFVNNY-NRWPTLRNAIVLPLRWL 494
HMPV     LRVHSTVLENN--DYPMYEVVLKLLGDTLRCIKLLINKNLENAAELYYIFRIFGHPMVDERDAMDAVKLNNEITKILRLESLTELRGAFILRI-IKGFVDNN-KRWPKIKNLKVLSKRWT 429
VSV      --R------------G-------IRFLHD---QIMSVKTVDLTLVIYGSFRHWGHPFIDYYTGLEKLHSQVTMKKDIDVSY-AKALASDLARIVLFQQFNDH-KKWFVNGDLLPHDHPFK 423
RABV     --P------------C-------ARRFFR---ALDQFDNIHDLVFVFGCYRHWGHPYIDYRKGLSKLYDQVHLKKMIDKSY-QECLASDLARRILRWGFDKY-SKWYLDSRFLARDHPLT 436
EBOV     LKP------------S---QAQKIREFHRT--LIRLEMTPQQLCELFSIQKHWGHPVLHSETAIQKVKKHATVLKALRPIVIFETYCVFKYSI-AKHYFDSQ-GSWYSVTSDRNLTPGLN 440
                                                 .    .    : :*** :    .   :       *                              *

MeV      NAQASGEGLT-HEQCVDNWKSFAGVRFGCFMPLSLDSDLTMYLKDKALAALQREWDSVYPKEFLRYDP-----------PKGTGSRRLVDVFLNDSSFDPYDMIMYVVSGAYLHDPEFNL 530
HPIV3    NAYGSNSAIS-YENAVDYYQSFIGIKFNKFIEPQLDEDLTIYMKDKALSPKKSNWDTVYPASNLLYRT-----------NASNESRRLVEVFIADSKFDPHQILDYVESGDWLDDPEFNI 538
HPIV1    NAQGSNSAIS-YECAVDNYSSFIGFKFLKFIEPQLDEDLTIYMKDKALSPRKAAWDSVYPDSNLYYKV-----------PESEETRRLIEVFINDNNFNPEDIINYVESGEWLNDDSFNI 538
Nipah    RLKNSGESLT-IDDCVKNWESFCGIQFDCFMELKLDSDLSMYMKDKALSPIKDEWDSVYPREVLSYTP-----------PKSTEPRRLVDVFVNDENFDPYNMLEYVLSGAYLEDDQFNV 537
Mumps    EMRHDNSELK-YEYVLKNWKSISMLRIHKCFDASPDEDLSIFMKDKAISCPKQDWMGVFRRSLIKQRYRD-----AHRPLPQSFNRRLLLNFLEDDRFDPIKELEYVTSGEYLRDPEFCA 548
PIV5     ELMNDNTEIS-YEFTLKHWKEVSLIKFKKCFDADAGEELSIFMKDKAISAPKQDWMSVFRRSLIKQRHQH-----HQVPLPNPFNRRLLLNFLGDDKFDPNVELQYVTSGEYLHDDTFCA 546
RSV      TYYKLNTYPSLLELTERDLIVLSGLRFYREFRLPKKVDLEMIINDKAISPPKNLIWTSFPRNYMPSHIQNYIEHEKLKFSESDKSRRVLEYYLRDNKFNECDLYNCVVNQSYLNNPNHVV 614
HMPV     MYFKAKNYPSQLELSEQDFLELAAIQFEQEFSVPEKTNLEMVLNDKAISPPKRLIWSVYPKNYLPETIKNRYLEETFNASDSLKTRRVLEYYLKDNKFDQKELKSYVVRQEYLNDKEHIV 549
VSV      SHVKENTWPT-AAQVQDFGDKWHELPLIKCFEIPDLLDPSIIYSDKSHSMNRSEVLKHVRMNP----------------NTPIPSKKVLQTMLDTKATNWKEFLKEIDE-KGLDDDDLII 525
RABV     PYIKTQTWPP-KHIVDLVGDTWHKLPITQIFEIPESMDPSEILDDKSHSFTRTRLASWLSENR----------------GGPVPSEKVIITALSKPPVNPREFLRSIDL-GGLPDEDLII 538
EBOV     SYIKRNQFPP-LPMIKELLWEFYHLDHPPLFSTKIISDLSIFIKDRATAVERTCWDAVFEPNVLGYNP-----------PHKFSTKRVPEQFLEQENFSIENVLSYAQKLEYLLPQYRNF 548
                                .     :      :     .*:: :  :         .                        .::    :     .             *
```

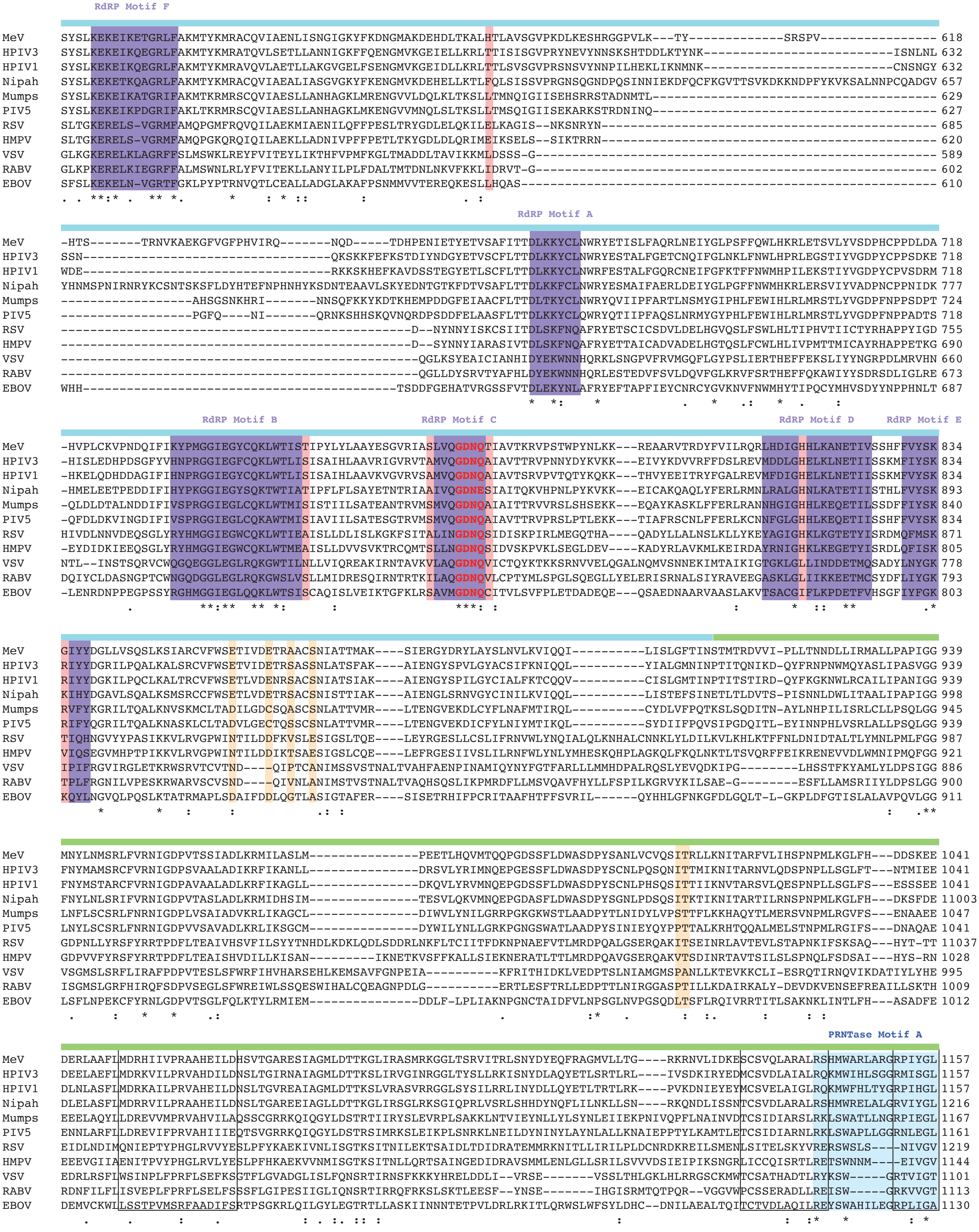
RdRP Motif F
RdRP Motif A
RdRP Motif B
RdRP Motif C
RdRP Motif D
RdRP Motif E
PRNTase Motif A
MeV
HPIV3
HPIV1
Nipah
Mumps
PIV5
RSV
HMPV
VSV
RABV
EBOV

```
                                                                                PRNTase Motif B                       PRNTase Motif C

MeV    EVPDVLESMRGHLIRRHETCVICE--CGSVNYGWFFVPSGCQLDDID--------KETSSLRVPYIGSTTDERTDMKLAFVRAPSR-SLRSAVRIATVYSWAYGDDDSSWNEAWL--LAR 1264
HPIV3  ETPDPLELLSGVVITGSEHCKICYSSDGTNPYTWMYLPGNIKIGSAE--------TGISSLRVPYFGSVTDERSEAQLGYIKNLSK-PAKAAIRIAMIYTWAFGNDEISWMEASQ--IAQ 1266
HPIV1  ETPDPLELLRGSFIEGSEVCKFCRSEGNNPMYTWFYLPDNIDLDTLS--------NGSPAIRIPYFGSATDERSEAQLGYVKNLSK-PAKAAIRIAMVYTWAYGTDEISWMEAAL--IAQ 1266
Nipah  EVPDALEAMVGRYITGSLECQICE--QGNTMYGWFFVPRDSQLDQVD--------KEHSSIRVPYVGSSTDERSDIKLGNVKRPTK-ALRSAIRIATVYTWAYGDNEECWYEAWY--LAS 1323
Mumps  ETPDPIELVHGCLIIGSDECEHCS--SGDDKFTWFFLPKGIRLDNDP--------ASNPPIRVPYIGSKTDERRVASMAYIKGASV-SLKSALRLAGVYIWAFGDTEESWQDAYE--LAS 1274
PIV5   ETPDPIEITAGALIVGSGYCEQCA--AGDNRFTWFFLPSGIEIGGDP--------RDNPPIRVPYIGSRTDERRVASMAYIRGASS-SLKAVLRLAGVYIWAFGDTLENWIDALD--LSH 1268
RSV    TSPSIMYTMDIKYTTST-------------------ISSGIIIE---KYNVNSLTRGERGPTKPWVGSSTQEKKTMPVYNRQVLTK-KQRDQIDLLAKLDWVYASIDNKDEFMEE--LSI 1314
HMPV   TSPSITTCMDVIYATSS-------------------HLKGIIIE---KFSTDRTTRGQRGPKSPWVGSSTQEKKLVPVYNRQILSK-QQREQLEAIGKMRWVYKGTPGLRRLLNK--ICL 1239
VSV    TVPHPLEML-GPQHRKETPCAPCNTS-G-----FNYVSVHCPDGIHD-------VFSSRGPLPAYLGSKTSESTSILQPWERESKVPLIKRATRLRDAISWFVEPDSKLAMTILSNIHSL 1207
RABV   TVPHPSEML-GLLPKSSISCTCGATGGG-----NPRVSVSVLPSFDQ-------SFFSRGPLKGYLGSSTSMSTQLFHAWEKVTNVHVVKRALSLKESINWFITRDSNLAQALIRNIMSL 1220
EBOV   TLPCMIEQFKVFWLKPYEQCPQCSNA--KQPGGKPFVSVAVKKHIVSAWPNASRISWTIGDGIPYIGSRTEDKIGQPAIKPKCPSA-ALREAIELASRLTWVTQGSSNSDLLIKP--FLE 1245
         *                                                                      :.** *.        :   .     :          *

                       PRNTase Motif D                                          PRNTase Motif E

MeV    QRANVSLEELRVITPISTSTNLAHRLRDRST-QVKYSGTSLVRV-ARYT-TISNDNLSFVI-SDKKVDTNFIYQQGMLLGLGVLETLFRLEKDTGSSNTVLHLHVE--TDCCVIPMIDHP 1378
HPIV3  TRANFTLDNLKILTPVATSTNLSHRLKDTAT-QMKFSSTSLIRV-SRFI-TMSNDNMSIKE-ANETKDTNLIYQQIMLTGLSVFEYLFRLKETTGHNPIVMHLHIE--DECCIKESFNDE 1380
HPIV1  TRANLSLENLKLLTPVSTSTNLSHRLRDTAT-QMKFSSATLVRA-SRFI-TISNDNMALKE-AGESKDTNLVYQQIMLTGLSLFEFNMRYKQGSLSKPMILHLHLN--NKCCIIESPQEL 1380
Nipah  QRVNIDLDVLKAITPVSTSNNLSHRLRDKST-QFKFAGSVLNRV-SRYV-NISNDNLDFRI-EGEKVDTNLIYQQAMLLGLSVLEGKFRLRLETDDYNGIYHLHVK--DNCCVKEVADVG 1437
Mumps  TRVNLTLEQLQSLTPLPTSANLVHRLDDGTT-QLKFTPASSYAF-SSYV-HISNDCQVLEI-DDQVTDSNLIYQQVMITGLALIETWNNPPINFSVYETTLHLHTG--SSCCIRPVESCV 1388
PIV5   TRVNITLEQLQSLTPLPTSANLTHRLDDGTT-TLKFTPASSYTF-SSFT-HISNDEQYLTI-NDKTADSNIIYQQLMITGLGILETWNNPPINRTFEESTLHLHTG--ASCCVRPVDSCI 1382
RSV    GTLGLTYEKAKKLFPQYLSVNYLHRLTVSSR-PCEFPASIPAYRTTNYHFDTSPINRILTE-KYGDEDIDIVFQNCISFGLSLMSVVEQFT---NVCPNRIILIPKLNEIHLMKPPIFTG 1429
HMPV   GSLGISYKCVKPLLPRFMSVNFLHRLSVSSR-PMEFPASVPAYRTTNYHFDTSPINQALSE-RFGNEDINLVFQNAISCGISIMSVVEQLT---GRSPKQLVLIPQLEEIDIMPPPVFQG 1354
VSV    TGEEWTKR-QHG--FKRTGSALHRFSTSRMSHGGFASQSTAAL-TRLM-ATTDTMRDL---G--DQNFDFLFQATLLYAQITT--T--VARDGWITSCTDHYHIA--CKSCLRPIEEIT 1310
RABV   TGPDFPLEEAPV---FKRTGSALHRFKSARYSEGGYSSVCPNLL-SHIS-VSTDTMSDL---TQDGKNYDFMFQPLMLYAQTWTSEL--VQRDTRLRDSTFHWHLR--CNRCVRPIDDVT 1328
EBOV   ARVNLSVQEILQMTPSHYSGNIVHRYNDQYS-PHSFMANRMSNSATRLI-VSTNTLGEFSGGGQSARDSNIIFQNVINYAVALFDIKFRNTEATDIQYNRAHLHLT---KCCTREVPAQY 1360
                          : .  **              :             :       :      :          : ::::*   :   .

MeV    RIPSSRKLELRAELCTNPLIYDNAPLIDRDATRLYTQ--SHRR-----H-----LVEFVTWSTPQLYHILAKSTALSMIDLVTKFEKDHMNEISALIGDDDINSFITEFLLIEPRLFTIY 1486
HPIV3  HINPESTLELIRYPESNEFIYDKDPLKDVDLSRLMVI--KDHS-----Y-----TIDMNYWDDTDIIHAISICTAITIADTMSQLDRDNLKEIIVIANDDDINSLITEFLTLDILVFLKT 1488
HPIV1  NIPPRSTLDLEITQENNKLIYDPDPLKDIDLELFSKV--KDVV-----H-----TIDMNYWSDDEIIRATSICTAMTIADTMSQLDRDNLKEMIALINDDDINSLITEFMVIDIPLFCST 1488
Nipah  QVDAELPIPEYTEVDNNHLIYDPDPVSEIDCSRLSNQ--ESKS-----R-----ELDFPLWSTEELHDALAKTVAQTVLEIITKADKDVLKQHLAIDSDDNINSLITEFLIVDPELFALY 1545
Mumps  VNPPLLPVPFINVPQMNKFVYDPEPLSLLEMEKIEDI--AYQT-----R-----IGGLDQIPLLEKIPLLAHLTAKQMVNSITGLDEATSIVNDAVVQADYTSNWISECCYTYIDSVFVY 1496
PIV5   LSEALTVKPHITVPYSNKFVFDEDPLSEYETAKLESL--SFQA-----Q-----LGNIDAVDMTGKLTLLSQFTARQIINAITGLDESVSLTNDAIVASDYVSNWISECMYTKLDELFMY 1490
RSV    ----DVDIHKLKQVIQKQHMFLPDKISLTQYVELFLSNKTLKSGSHVNSNLILAHKISDYFHNTYI---LSTNLAGHWILIIQ-----LMKDSKGIFEKDWGEGYITDHMFINLKVFFNA 1537
HMPV   ----KFNYKLVDKITSDQHIFSPDKIDMLTLGKMLMPTIKGQ------KTDQFLNKRENYFHGNNLIESLSAALACHWCGILT-----EQCIENNIFKKDWGDGFISDHAFMDFKIFLCV 1459
VSV    LDS--------------SMDYTPPDVSHVLKTWRNGEGSWGQEI----KQIYPLEGNWKNLAPAEQSYQVGRCIGFLYGDLA------------YRKSTHAEDSS----------LF--- 1387
RABV   LET--------------SQIFEFPDVSKRISRMVSGAVPHFQRL----PDIRLRPGDFESLSGREKSHHIGSAQGLLYSILV------------AIHDSGYNDGT----------IF--- 1405
EBOV   LTYTSTLDLDLTRYRENELIYDSNPLKGGLNCNISFDNPFFQG-----K-----RLNIIEDDLIRLPHLSGWELAKTIMQSIIS---DSNNSSTDPISSGETRSFTTHFLTYPKIGLLYS 1467
                           :   :                                                                  .   .                            .           .

MeV    LGQCAAINWAFDVHYHRPSGKYQ--------MGELLSSFLSRMSKGVFKVLVNALSHPKIYKKFWHCGIIEPIHGPSLDA----QNLHTTVCNMVYTCYMTYLDLLLNE--ELEEFTFLL 1592
HPIV3  FGGLLVNQFAYTLYSLKIEGRDL--------IWDYIMRTLRDTSHSILKVLSNALSHPKVFKRFWDCGVLNPIYGPNTAS----QDQIKLALSICEYSLDLFMREWLNG----VSLEIYI 1592
HPIV1  FGGILINQFAYSLYGLNIRGRDE--------IWGYVIRIIKDTSHAVLKVLSNALSHPKIFKRFWDAGVVEPVYGPNLSN----QDKILLAISVCEYSVDLFMRDWQEG----IPLEIFI 1592
Nipah  LGQSIAIKWAFEIHHRRPRGRHT--------MVDLLSDLISNTSKHTYKVLSNALSHPRVFKRFVNCGLLLPTQGPYLHQ----QDFEKLSQNLLVTSYMIYLMNW--C--DFKKYPFLI 1649
Mumps  SGWALLLELSYQMYYLRIQGIQG--------ILDYVYMTLRRIPGMAITGISSTISHPRILRRCINLDVIAPINSPHIAS----LDYTKLSIDAVMWGTKQVLTNISQG----IDYEIVV 1600
PIV5   CGWELLLELSYQMYYLRVVGWSN--------IVDYSYMILRRIPGAALNNLASTLSHPKLFRRAINLDIVAPLNAPHFAS----LDYIKMSVDAILWGCKRVINVLSNG----GDLELVV 1594
RSV    YKTYLLCFHKGY--GKAKLECDM--------NTSDLLCVLELIDSSYWKSMSKVFLEQKVIKYILSQDASLHRVKGCHS-------------------F----KLWFLKRLNV------- 1617
HMPV   FKTKLLCSWGSQ--GKNIKDEDI--------VDE-SIDKLLRIDNTFWRMFSKVMFEPKVKKRIMLYDVKFLSLVGYIG-------------------F----KNWFIEQLRS------- 1538
VSV    ---------PLSI-QGRIRGRGFLKGLLDGLMRASCCQVIHRRSLAHLKRPANAVYGGLIY-LIDKLSVSPPFLSL---------------------------TRSGP-IRDELETIPHKI 1469
RABV   ---------PVNI-YGKVSPRDYLRGLARGVLIGSSICFLTRMTNININRPLELVSGVISY-ILLRLDNHPSLYIM---------------------------LREPS-LRGEIFSIPQKI 1487
EBOV   FGAFVSYYLGNTILRTKKLTLDN--------FLYYLTTQIHNLPHRSLRILKPTFKHASVMSRLMSIDPHFSIYIGGAAGDRGLSDAARLFLRTSISSFLTFVKEWIINRGTIVPLWIVY 1579
                                          :                .                  .

MeV    CESDEDVVPDRFDNI-----QAKHLCVLADLYCQPGTCPPIRGLRPVE-KCAVLTDHIKAEARLSP------AGSSWNINPIIVDHYSCSLTYLRR-----G--SIKQIR-----LRVD- 1687
HPIV3  CDSDMEVANDRKQAF-----ISRHLSFVCCLAEIASFGPNLLNLTYLE-RLDLLKQYLELNIKEDP------TLKYVQISGLLIKSFPSTVTYVRK-----T--AIKYLR-----IRGI- 1687
HPIV1  CDNDPHIAEMRKLSF-----LARHLAYLCSLAEIAKEGPKLESMTSLE-RLESLKEYLELTFLDDP------ILRYSQLTGLVIKIFPSTLTYIRK-----S--SIKVLR-----VRGI- 1687
Nipah  AEQDETVINLREDII-----TSKHLCVIIDLYANHHKPPWIIDLNPQE-KICVLRDFISKSRHMDT------SSRSWNTSDLDFVIFYASLTYLRR-----G--IIKQLR-----IRQV- 1744
Mumps  PSESQLTLSDRVLNL-----VARKLSLLAIIWANYNYPPKVKGMSPED-KCQALTTHLIQTVEYVEHIQIEKTNIRRMIIEPKLTAYPSNLFYLSR-----K--LLNAIR-----DSEE- 1701
PIV5   TSEDSLILSDRSMNL-----IARKLTLLSLIHHNGLELPKIKGFSPDE-KCFALTEFLRKVVNSGLSSIENLSNFMYNVENPRLAAFASNNYYLTR-----K--LLNSIR-----DTES- 1695
RSV    ---------------------------------AEFTVCPWVVNIDYHPTHMKAILTYIDLVR-MGLINID--RIHIKNKHKFNDEFYTSNLFYINYNFSDNTHLLTKHIRIANSELENN- 1701
HMPV   ---------------------------------AELHEIPWIVNAEGDLVEIKSIKIYLQLIE-QSLFLR----I-------------------TVLNYTDMAHALTRLIRKKLM------ 1596
VSV    PTS--YPTSNRDMGVIVRNY----FKYQCRLIEKGKYRS-------HYSQLWLFSDVLSIDFI-------------------GPFSISTTLLQILY-----KPFLSGKDKNELRELANLS 1552
RABV   PAA--YPTTMKEGNRSILCYLQHVLRYEREIITA---SP-------ENDWLWIFSDFRSAKMTY-----------------LSLITYQSHLLLQRV-----ERNLSKSMRDNLRQLSSLM 1573
EBOV   PLEGQNPTP--VNNF-----LYQIVELLV---HD------------SS-RQQAFKTTISDHVHPHD-------NLVLYTCKSTASNFFHASLAYWRS-----R--HRNSNRKYLARDSST- 1661
                                                            :                                                   :

MeV    ---------P--------GFIF---DALA--------------EVNVSQPKV-------G---SNNISNMS-I---KDFRPPHDDVAKLLKDINT------------------------ 1734
HPIV3  -------SPP--------EIID---DWDPI---ED--------ENMLDNIVK-----SISDNCNKDNKGNK-INNFWGLALKNYQVLKIRSITSD-SDN-------------------NN 1752
HPIV1  -------GIP--------EVLE---DWDPD---AD--------SMLLDNITA-----EVQHNIP--LKKNE-RTPFWGLRVSKSQVLRLRGYEEI-KRE-------------------ER 1750
Nipah  ---------T--------EVVD---TTTML---RD--------NIIVENPPI-----KTG---VLDIRGCI-I---YNL--------EEILSMNTK------------------------ 1789
Mumps  -------GQF--------LIAS---YYNSF-GYLE--------PILMESK-------IFSLSSSESASLTE-FDFILNLELSEASLEKYSLPSL------------------------- 1760
PIV5   -------GQV--------AVTS---YYESL-EYID--------SLKLTPH-------VPGTSCIEDDSLCT-NDYIIWIIESNANLEKYPIPNS------------------------- 1754
RSV    -------------------Y---NKLYHPTPETLE--------NILANPIKS-NDKKTLNDYCIGKNVDSI-MLPLLSN----KKLIKSSAMIR----TN---YSKQDLYNLFPMVVIDR 1778
HMPV   --------------------------------CD---------NALLTPI----------------------SSPMVNL----TQVID------------------PTTQLDYFPKITFER 1632
VSV    SLLRSGEGWEDIHVK----FFT----KDI---------------LLCPEEIRHACK-----FGIAKDNNKDMSYPPWGR-ESRG-TI---------------------------------- 1609
RABV   RQVLGGHGEDTLESDDNIQRLL----KDSL---RR--------TRWVDQEVRHAARTMTGDYSPNKKVSRKVGCSEWVC-SAQQ-VA--------------------------------- 1643
EBOV   -------GSSTNNSDGHIERSQEQTTRDPHDGTERNLVLQMSHEIKRTTIPQENTHQGPSFQSFLSDSACGTANPKLNFDRSRHNVK-FQDHNSASKREGHQIISHRLVLPFFTLSQGTR 1773
```

```
MeV     --------SKHNLP----ISGGSLANYE-------------IHAFRR-IGLNSSACYKAVEISTLIRRCLEPGEDGLFLGEGSGSMLITYKEILKLNKCF-YNSGVSANSRSG------- 1820
HPIV3   GL------DVSTGG----LTLPQGGNYL-------------SHQLRL-FGINSTSCLKALELSQILMKEVNKDKDRLFLGEGAGAMLACYDATLGPAVNY-YNSGLNITDVIG------- 1840
HPIV1   --------GRSGVG----LTLPFDGRYL-------------SHQLRL-FGINSTSCLKALELTYLLNPLVNKDKDRLYLGEGAGAMLSCYDATLGPCMNY-YNSGVNSCDLNG------- 1836
Nipah   --------SSSKKI----FNLNSKPSVE-------------NHKYRR-IGLNSSSCYKALNLSPLIQRYLPSGAQRLFIGEGSGSMMLLYQSTLGQSISF-YNSGIDGDYIPG------- 1875
Mumps   --------LMTAEN----MDNPFPQPPL-------------HHVLRP-LGLSSTSWYKTISVLNYISHMKISDGAHLYLAEGSGASMSLIETFLPGETIW-YNSLFNSGENPP------- 1846
PIV5    --------PEDDSN----FHNFKLNAPS-------------HHTLRP-LGLSSTAWYKGISCCRYLERLKLPQGDHLYIAEGSGASMTIIEYLFPGRKIY-YNSLFSSGDNPP------- 1840
RSV     IIDHSGNTAKSNQLYTTTSHQISLVHNST----SLYCMLPWHHINRFNFVFSSTGCKISIEYILKD-LKIKDPNCIAFIGEGAGNLLLRTVVELHPDIRYIYRSLKDCNDHSL------- 1886
HMPV    LKNYDTS------------SNYAKGKLTR----NYMILLPWQHVNRYNFVFSSTGCKVSLKTCIGKLMKDLNPKVLYFIGEGAGNWMARTACE-YPDIKFVYRSLKDDLDHHY------- 1728
VSV     ---------------TTIPVYYTTTPYPKMLEMPPRIQNPLLSGIRL-GQLPTGAHYKIRSILHG---MGIHYRDFLSCGDGSGGMTAALLREN-VHSRGIFNSLLELSGSVM------- 1702
RABV    ---------------VSTSANPAPVSELDIRALSKRFQNPLISGLRV-VQWATGAHYKLKPILDD---LNVFPSLCLVVGDGSGGISRAVLNMF-PDAKLVFNSLLEVNDLMA------- 1736
EBOV    QL------TSSNES----QTQDEISKYLR----QLRSVIDTTVYCRF-TGIVSSMHYKLDEVLWEIESF--KSAVTLAEGEGAGALLLIQKYQ--VKTLF-FNTLATESSIESEIVSGMT 1873
                                                     *      :                          .:*:*                 :.:

MeV     -QRELAPYPSEVG------LVEHRMGV----GNIVKVLFNGRPEVTWVGSIDCFNFIVSN--IPTSSVGFIHSDIETLPNKDTIEKLEELAAILSMA---LLLGKIGSILVIK-LMPFSG 1923
HPIV3   -QRELKIFPSEVS------LVGKKLGNVTQILNRVKVLFNGNPNSTWIGNMECESLIWSE--LNDKSIGLVHCDMEGAIGKSEETVLHEHYSVIRIT---YLIGDDDVVLVSK-IIPTIT 1947
HPIV1   -QRELNIYPSEVA------LVGKKLNNVTSLCQRVKVLFNGNPGSTWIGNDECETLIWNE--LQNNSIGFIHCDMEGGEHKCDQVVLHEHYSVIRIA---YLVGDKDVILVSK-IAPRLG 1943
Nipah   -QRELKLFPSEYS------IAEEDPSLAGKLKGLVVPLFNGRPETTWIGNLDSYEYIINR--TAGRSIGLVHSDMESGIDKNVEEILVEHSHLISIA---INVMMEDGLLVSK-IAYTPG 1982
Mumps   -QRNFAPLPTQFIESVPYRLIQAGIAAGSGVVQSFYPLWNGNSDITDLSTKTSVEYIIHK--VGADTCALVHVDLEGVPGSMNSMLERAQVHALLIT---VTVLKPGGLLILK-ASWEPF 1959
PIV5    -QRNYAPMPTQFIESVPYKLWQAHTDQYPEIFEDFIPLWNGNAAMTDIGMTACVEFIINR--VGPRTCSLVHVDLESSASLNQQCLSKPIINAIITA---TTVLCPHGVLILK-YSWLPF 1953
RSV     --------PIEFLR-----LYNGH-INIDYGEN-------LTIPATDATNNIHWSYL-HI--KFAEPISLFVCDAELSVTVNWSKIIIEWSKHVRKCKYCSSVNKC-M-LIVK-YHAQDD 1979
HMPV    --------PLEYQR-----VIGELSRIIDSGEG-------LSMETTDATQKTHWDLI-HR--VSKDALLITLCDAEFKDRDDFFKMVILWRKHVLSCRICTTYGTD-LYLFAK-YHAKDC 1823
VSV     --RGASPEPPSALET----LGGD-------K-SRCVNGETCWEYPSDLCDPRTWDYFLRLKAGLGLQIDLIVMDMEVRDSSTSLKIETNVRNYVHR------ILDEQGVLIYKTYGTYIC 1802
RABV    --SGTHPLPPSAIMR----GGND-------IVSRVIDLDSIWEKPSDLRNLATWKYFQSVQKQVNMSYDLIICDAEVTDIASINRITLLMSDFAL-------SIDGPLYLVFKTYGTMLV 1836
EBOV    TPRMLLPVMSKFHN-----------------DQIEIILNNSASQITDITNPTWFKDQRAR--L-PKQVEVITMDAETTENINRSKLYEAVYKLILHH---IDPSVLKA-VVLK-VFLSDT 1968
                  .                                  :                       .   * *                                  :.  *

MeV     DFVQGFISYVGSHYREVNLVYPRYSNFISTESYLVMTDLKANRLMNPEKIKQQIIESSVRTSPGLIGHILSIKQLSCIQAI--VGGAVSR-GDI-----NPILKKLTPIEQVLISCGLAI  2035
HPIV3   PNWSRILYLYKLYWKDVSIISLKTSNPASTELYLISKDAYCTIMEPSEVVLSKLKRLSLLEENNLLKWIILSKKRNNEWLHHEIKE-----G-------ERDYGVMRPYHMALQIFGFQI  2055
HPIV1   TDWTKQLSLYLRYWRDVSLIVLKTSNPASTEMYLISKDPKSDIIEDSNTVLANLLPLSKEDSIKIEKWILVEKAKVHDWIVRELKE-----G-------SASSGMLRPYHQALQIFGFEP  2051
Nipah   FPISRLFNMYRSYFGLVLVCFPVYSNPDSTEVYLLCLQKTVKTIIPPQRVLEHSDLHDEVNDQGITSVIFKIKNSQSKQFHEDLKKYYHI-DQP-----FFVPTKITSDEQVLLQAGLKL  2096
Mumps   NRFSFLLTILWQFFSTIRILRSSYSDPNNHEVYIIATLAVDPTTSS---FTTALNRARTLNEQGFS---LIPPELVSEYWRRRVEQGQIIQDRIDKVISECVRDQYLADNNIILQAGGTP  2073
PIV5    TRFSTLITFLWCYFERITVLRSTYSDPANHEVYLICILANNFAFQT---VSQATGMAMTLTDQGFT---LISPERINQYWDGHLKQERIVAEAIDKVVLGENALFNSSDNELILKCGGTP  2067
RSV     ID--FKL----DNITILKTYVCLGSKLKGSEVYLVLTIGPANIFPVFNVV-QNAKLIL-SRTKNFI----MPKK---------ADK-E--------------------------------  2045
HMPV    NV--KLP----FFVRSVATFIMQGSKLSGSECYILLTLGHHNSLPCHGEI-QNSKMKI-AVCNDFY----AAKK---------LDN-K--------------------------------  1889
VSV     ESEKNAVTILGPMFKTVDLVQTEFSSSQTSEVYMVCKGLKKLIDEPN-P-DWSS-----INESWKNLYAFQSSEQEFARA-KKVSTYFTLTGIPS----QFIPDPFVNIETMLQIFGVPT  1910
RABV    NPNYKAIQHLSRAFPSVTGFITQVTSSFSSELYLRFSKRGKFFRDAE-YLTSST-----LREMSLVLFNCSSPKSEMQRA-RSLNYQDLVRGFPE----EIISNPYNEMIITLIDSDVES  1945
EBOV    EGMLWLNDNLAPFFATGYLIKPITSSARSSEWYLCLTNFLSTTRKMPHQN--------HLSCKQVILTALQLQIQRSPYWLSHLTQYADCELHLS----------------YIRLGFPS  2063
                                :.    * *:

MeV     NGPKLCKELIHHDVA-SGQDGLLNSIL----ILYRELARF--K-DNQRSQQGMFHAY--PVLVSSRQRELVSRITRKFWGHILLYS--GN-RKLINRFIQNLK-----SGYLVLDLH---  2134
HPIV3   NLNHLAKEFLSTPDL-TNINNIIQSFQRTIKDVLFEWINITHD-DKRHKLGGRYNIF--PLKNKGKLRLLSRRLVLSWISLSLSTRLLTG-RFPDEKFEHRAQ-----TGYVSLADT---  2162
HPIV1   NLNKLCRDFLSTLNI-VDTKNCIITFDRVLRDTIFEWTRIKDV-DKKLRLTGKYDLY--PLRDSGKLKVISRRLVISWIALSMSTRLVTG-SFPDMKFESRLQ-----LGIVSISSR---  2158
Nipah   NGPEILKSEISYDIG-SDINTLRDTII----IMLNEAMNY--F-DDNRSPSHHLEPY--PVLERTRIKTIMNRVTKKVIVYSLIKFKDTK-SSELYHIKNNIR-----RKVLILDFR---  2197
Mumps   STR----KWLDLPDY-PSFNELQSEMARLITIHLKEVVEILKG-QSSDHDTLLFTSY--NVGPLGKINTILRLIVERILMYTVRNWCILP-TQTRLTLRQSIE-----LGEFRLRDVITP  2179
PIV5    NAR----NLIDIEPV-ATFIEFEQLICTMLTTHLKEIIDITRS-GTQDYESLLLTPY--NLGLLGKISTIVRLLTERILNHTIRNWLILP-PSLRMIVKQDLE-----FGIFRITSILNS  2173
RSV     SIDANIKSLIPFLCYPITKKGIN-TAL---------------------SKLKSVVSGDILSYSIAGRNEVFSNK----LINHKHMNI-----LKWFNHVLNFRS------T----------  2119
HMPV    SIEANCKSLLSGLRIPINKKELD-RQR---------------------RLLT--LQSNHSSVATVGGSKIIESK----WLTNKASTI-----IDWLEHILNSPK------G----------  1961
VSV     GVSHAAALKSSDRP------ADLLTIS----LFYMAII----SYYNINHIRVGPIPP--NPP-SDGI---A----------QNVGIAITGISFWLSLMEKDIPLYQQCLAV---------  1991
RABV    FLVHKMVDDLELQR------GTLSKVA----IIIAIMIVFSNRVFNVSKPLTDPSFY--PPS-DPKI---L----------RHFNICCST-MMYLSTALGDVPSFARLHDL---------  2029
EBOV    -LE---KVLYHRYNLVDSKRGPLVSITQHLAHLRAEIRELTND-YNQQR-QSRTQTYHFIRTAKGRITKLVNDYLKFFLIVQALKH--NG-T-WQAEFKKLPELISVCNRFYHIRDC---  2170

MeV     -QNI--------FVKNLSKSEKQIIMTGGLKREWVFKVTV----------------KETKEWYKLVGYSALIKD------------------------------------------  2183
HPIV3   -DLESLKL----LSKNIIKNYRECI---GS---ISYWFLT----------------KEIKILMKLIGGAKLLGIPRQYKEPEEQLL-EHYNQHDEFDID--------------------  2233
HPIV1   -EIKNLRV----ISKIVIDKFEDII---HS---VTYRFLT----------------KEIKILMKILGAVKLFGARQSMSVDITNI-----DTSDSIQ----------------------  2223
Nipah   -SKL--------MTKTLPKGMQERREKSGFKEVWIVDLSN----------------REVKIWWKIIGYISII------------------------------------------------  2244
Mumps   MEILKLSPNRKYLKSALNQSTFNHLMGE-----TSDILLNR----------------AYQKRIWKAIGCVIYCFGLLTPDVEDSERIDID-NDTPDYDIHG--DII---------------  2261
PIV5    DRFLKLSPNRKYLIAQLTAGYIRKLIEG-----DCNIDLTR----------------PIQKQIWKALGCVVYCHDPMDQRE-STEFIDININEEIDRGIDG--EEI---------------  2255
RSV     ----ELNYNHLYMVESTYPYLS-----------ELLNSLTT----------------NELKKLIKITGSLLYNFHNE-----------------------------------------  2165
HMPV    ----ELNYDFFEALENTYPNMI-----------KLIDNLGN----------------AEIKKLIKVTGYMLVSKK-------------------------------------------  2005
VSV     ------------IQQSFPIRWEAVSVKGGYKQKWSTRGDG-LPKDTRISDSLAPIGNWIRSLELVRNQVRLNPFNEILFNQLCRTVDNHLKWSNLR---RNTGMIEWINRRISKEDRSIL  2095
RABV    ------------YNRPITYYFRKQVIRGNVYLSWSWSNDTSVFKRVACNSSLSLSSHWIRLIYKIVKTTRLVGSIKDLSREVERHLHRYNRWITLEDIRSRSSLLDYS----------CL  2127
EBOV    ------NCEERFLVQT---------------LYLHRMQD----------------SEVKLIERLTGLLSLFPDGLYRFD-----------------------------------------  2212
                                                                   :

MeV     -------------- 2183
HPIV3   -------------- 2233
HPIV1   -------------- 2223
Nipah   -------------- 2244
Mumps   -------------- 2261
PIV5    -------------- 2255
RSV     -------------- 2165
HMPV    -------------- 2005
VSV     MLKSDLHEENSWRD 2109
RABV    -------------- 2127
EBOV    -------------- 2212
```
