# Supplementary figures and images for: Therapeutic targeting of measles virus polymerase with ERDRP-0519 suppresses all RNA synthesis activity
Source: PLoS Pathog. 2021 Feb 23;17(2):e1009371. doi: 10.1371/journal.ppat.1009371 (PMC7935272; doi:10.1371/journal.ppat.1009371)

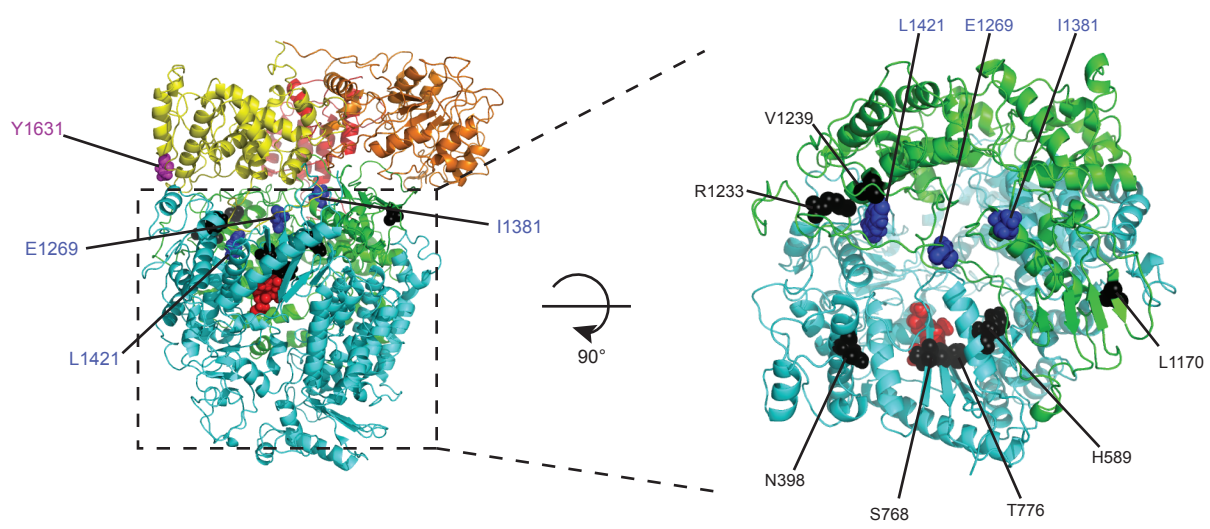

Supplement: S2 Fig — Location of ERDRP-0519 resistance mutations (black) in comparison with escape sites from an RSV L capping inhibitor (blue) and RSV L blocker AZ-27 (magenta). Side views of the MeV L complex and top view of the RdRP and capping domains only are shown. The GDNQ catalytic center is highlighted in red. (PDF) [file ppat.1009371.s002.pdf]

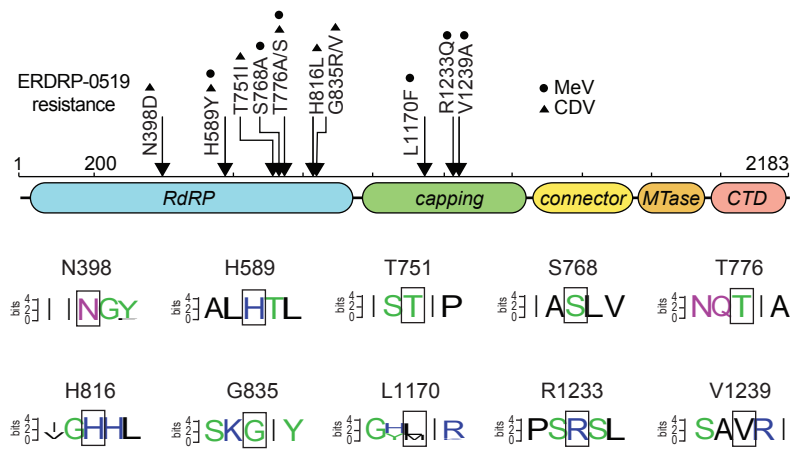

Supplement: S3 Fig — Consensus sequences for residues identified in previous resistance profiling studies in MeV and CDV against ERDRP-0519. All complete L protein sequences in the NCBI virus database [67] for MeV (332 sequences), CDV (178 sequences), and peste des petits ruminants virus (PPRV; 55 sequences; S10 Data) were aligned with Clustal-Omega [66] and consensus sequences were generated using WebLogo [68]. The specific residue involved in resistance to ERDRP-0519 is boxed. (PDF) [file ppat.1009371.s003.pdf]

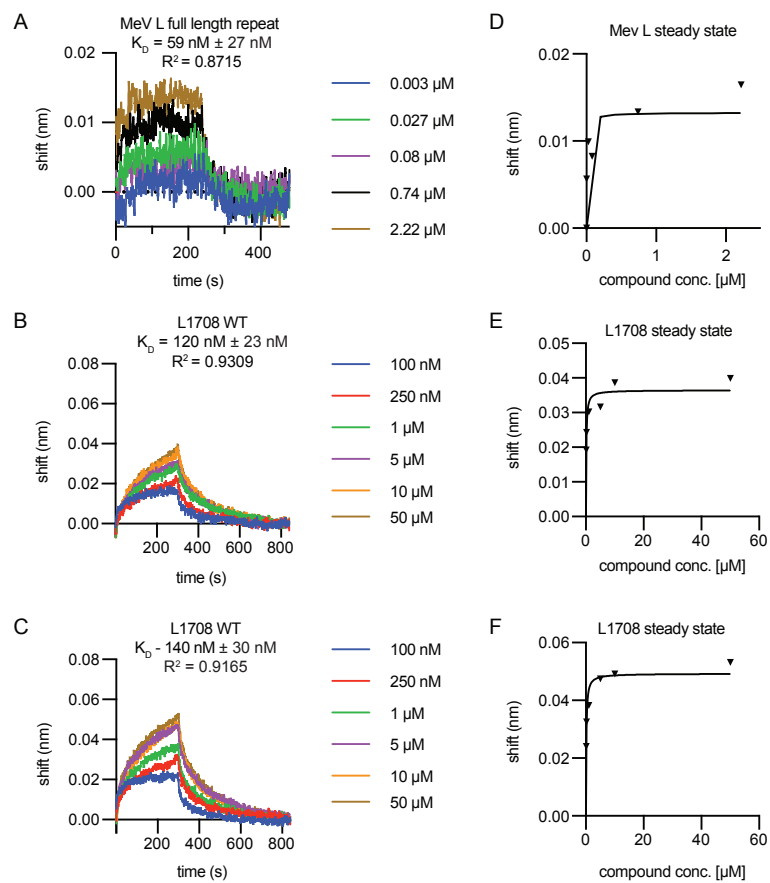

Supplement: S5 Fig — A-C) Experimental repeats of BLI of ERDRP-0519 and purified standard (WT) full-lengths MeV L (A) and MeV L1708 (B-C). KD values and goodness of fit are shown for each construct. D-F) Concentration-dependent steady-state BLI sensor response signals from results shown in (A-C) were plotted for full-lengths L (D) and MeV L1708 (E-F). (PDF) [file ppat.1009371.s005.pdf]

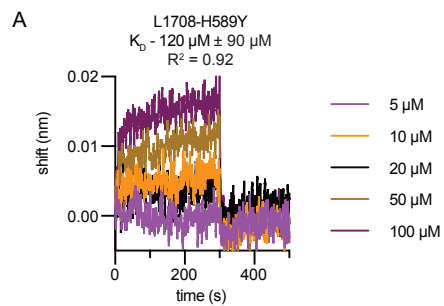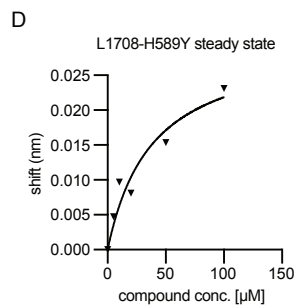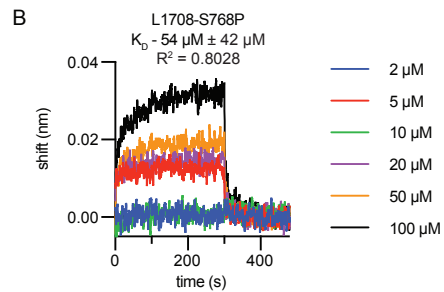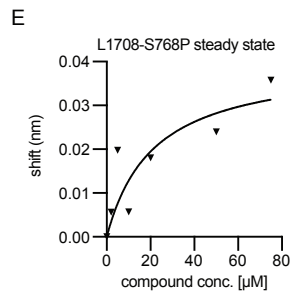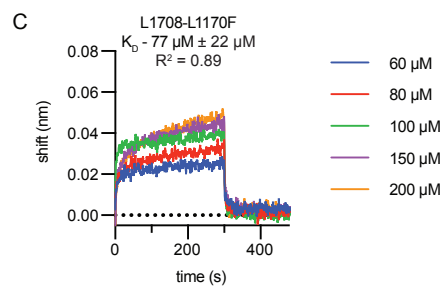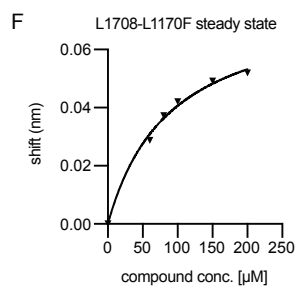

Supplement: S6 Fig — A-C) Experimental repeats of BLI of ERDRP-0519 and purified MeV L1708 resistance mutations H589Y(A), S768P (B) and L1170F (C). KD values and goodness of fit are shown for each construct. D-F) Concentration-dependent steady-state BLI sensor response signals from results shown in (A-C). (PDF) [file ppat.1009371.s006.pdf]

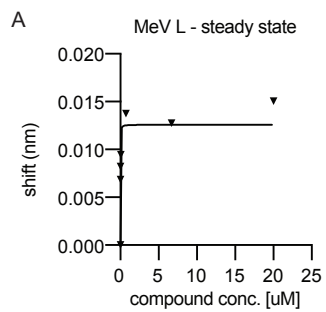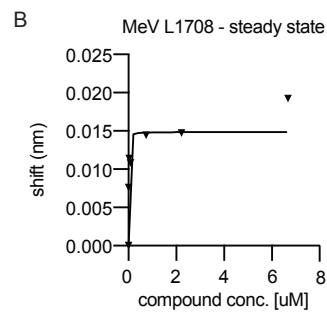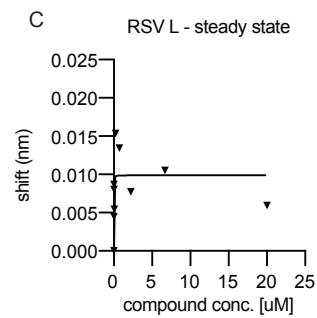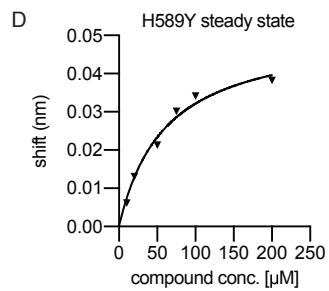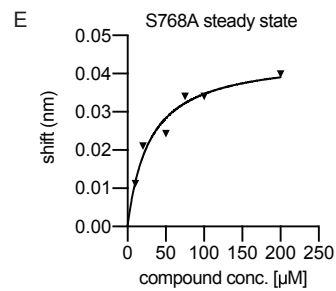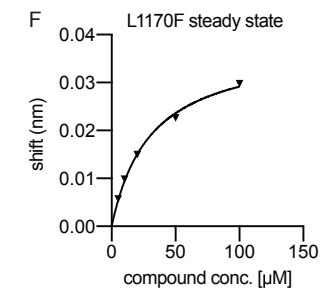

Supplement: S7 Fig — A-F) Concentration-dependent steady-state BLI sensor response signals were plotted for the different L populations (full-length MeV L (A), MeV L1708 (B) RSV L (C) and MeV L1708 carrying resistance mutations H589Y(D), S768P (E) and L1170F (F). (PDF) [file ppat.1009371.s007.pdf]

**primer extension**

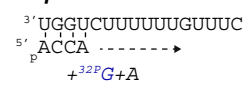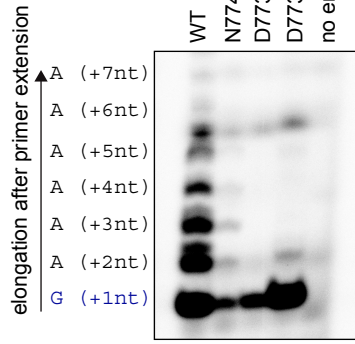

Supplement: S8 Fig — Purified recombinant WT MeV L-P complexes or complexes harboring LD773A, LN774A or LD773A N774A substitutions in the highly conserved RdRP GDN motif were incubated with a 16-nt RNA template, a 5’-phosphorylated 4-nt primer, and the specified NTPs to assess primer extension. RNA products were separated by 7M urea 20% polyacrylamide gel electrophoresis and visualized by autoradiography. Representative autoradiogram is shown (n = 3). As a control for background signal due to unincorporated isotope, a reaction with no enzyme is included. (PDF) [file ppat.1009371.s008.pdf]

Figure S9. Autoradiogram of *de novo* RdRP assay with ERDRP-0519az

*de novo* RNA synthesis

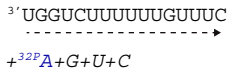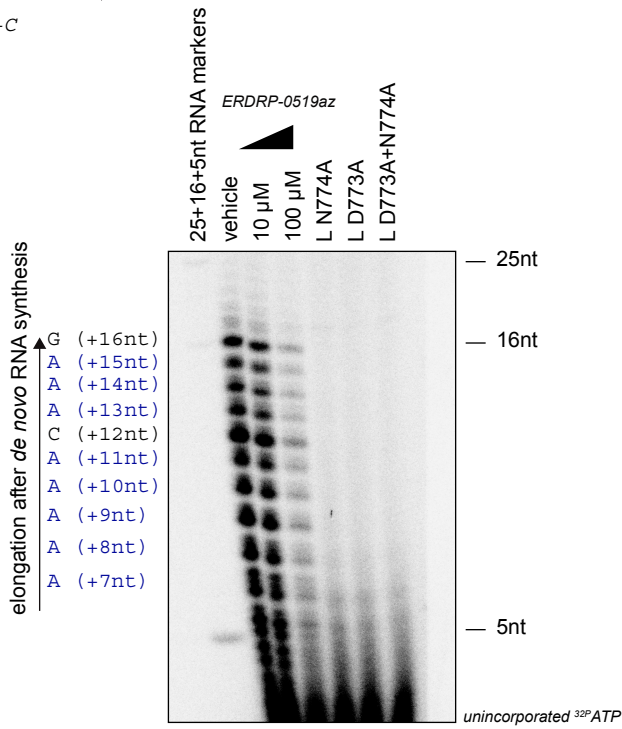

Supplement: S9 Fig — Purified recombinant standard MeV L-P complexes or complexes harboring substitutions in the RdRP GDN motif LD773A, LN774A or LD773A N774A as specified were incubated with a 16-nt RNA template to assess the effect of ERDRP0519az on de novo RNA synthesis. RNA products were separated by 7M urea 20% polyacrylamide gel electrophoresis and visualized by autoradiography. A representative autoradiogram is shown (n = 2). (PDF) [file ppat.1009371.s009.pdf]

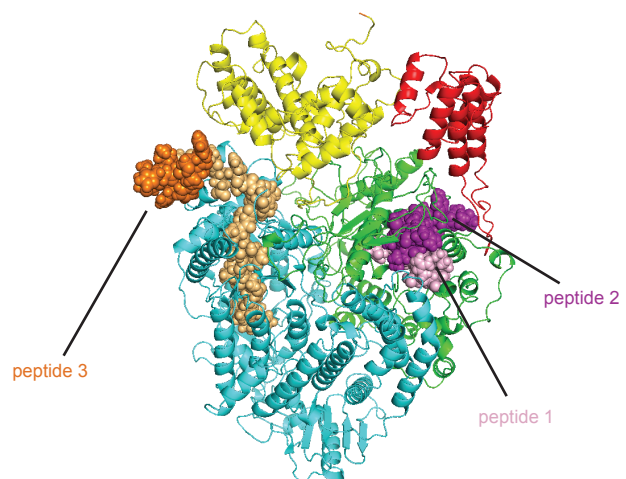

Supplement: S10 Fig — Peptides 1 and 2 are shown in pink and purple, respectively. Crosslinking-identified peptide 3 (orange spheres) is also highlighted. Residues directly engaged by ERDRP-0519az are shown as dark orange spheres. (PDF) [file ppat.1009371.s010.pdf]

precursor compound 1

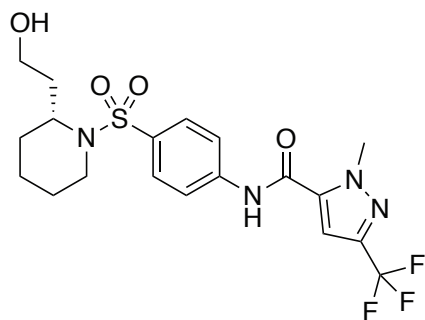

NaH  
1-azido-4-(bromomethyl)benzene  
DMF  
0°C-RT, 5 h

ERDRP-0519<sub>az</sub>

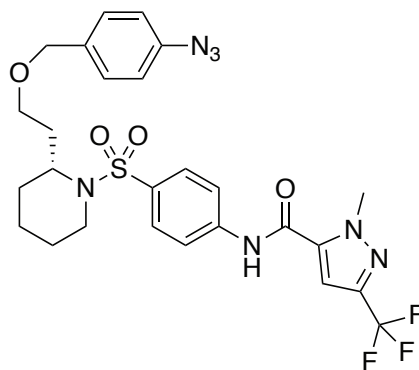

Supplement: S15 Fig — (PDF) [file ppat.1009371.s015.pdf]

**S1 Dataset.** Source and biological repeats from figure 2A.

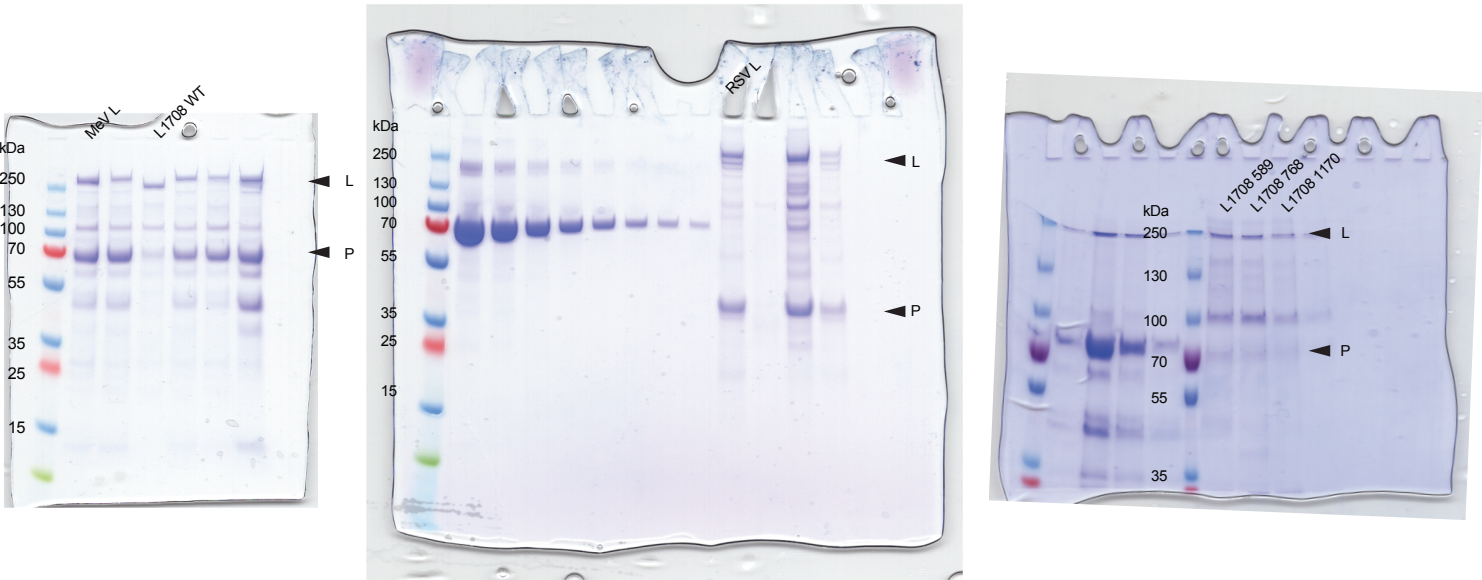

Supplement: S1 Data — (PDF) [file ppat.1009371.s018.pdf]
