# Supplementary material for: Therapeutic targeting of measles virus polymerase with ERDRP-0519 suppresses all RNA synthesis activity
Source: PLoS Pathog. 2021 Feb 23;17(2):e1009371. doi: 10.1371/journal.ppat.1009371 (PMC7935272; doi:10.1371/journal.ppat.1009371)
Supplement: S4 Fig — A-D) Antiviral synergy scores mapped to the dose response of ERDRP-0519 vs GHP-88309 measuring antiviral potency (n = 4) or cytotoxicity (n = 5). Synergy scores plotted over the matrix of different compound concentrations tested (blue = synergy, red = antagonism) are shown in (A). The sum of synergy and antagonism observed for ERDRP-0519 and GHP-88309 against MeV was positive (26.64; synmax = 42.1, antmax = -0.89), indicating synergy, but drug combinations did not increase cytotoxicity (B). Data analysis according to the HSA model [69] for antiviral (C) and cytotoxic (D) effects. E-F) Matrices showing the synergy scores for antiviral efficacy (E) or cytotoxicity (F) over a range of different compound concentrations. (PDF) [file ppat.1009371.s004.pdf]

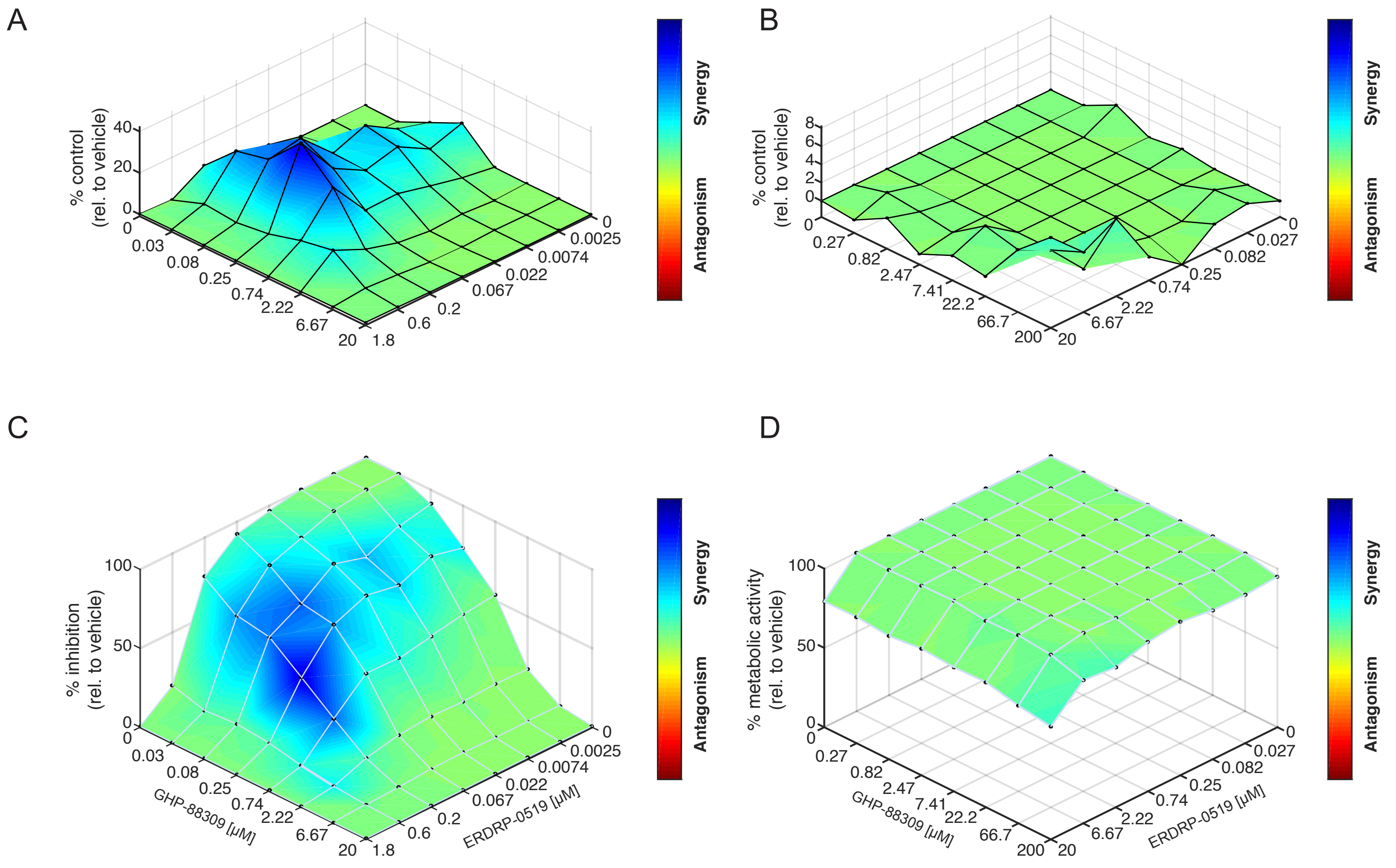

E

GHP-88309 [µM]

ERDRP-0519 [µM]	0.03	0.08	0.25	0.74	2.22	6.67	20
0.0025	0 +/-0 ***	11 +/-17	9 +/-25	3 +/-20	-1 +/-5	0 +/-1	0 +/-1
0.0074	3 +/-6	20 +/-17	18 +/-21	6 +/-22	0 +/-5	0 +/-2	0 +/-1
0.022	10 +/-8	14 +/-17	14 +/-17	10 +/-12	-1 +/-6	-1 +/-2	0 +/-1
0.067	17 +/-18	29 +/-22	22 +/-18	10 +/-20	1 +/-6	0 +/-2	0 +/-1
0.2	23 +/-19	27 +/-14 *	42 +/-11 *	28 +/-9 *	6 +/-1 *	1 +/-0 *	0 +/-1
0.6	5 +/-4	4 +/-5	6 +/-4	10 +/-2 *	13 +/-1 **	2 +/-1 *	0 +/-1
1.8	0 +/-0	0 +/-0	0 +/-1	0 +/-1	0 +/-0	1 +/-0	0 +/-1

HSA synergy and antagonism

F

GHP-88309 [µM]

ERDRP-0519 [µM]	0.27	0.82	2.47	7.41	22.2	66.7	200
0.027	0 +/-0 ***	0 +/-0 ***	0 +/-0 ***	0 +/-0 ***	0 +/-0 ***	1 +/-3	1 +/-5
0.082	0 +/-0 ***	0 +/-0 ***	0 +/-0 ***	0 +/-0 ***	0 +/-0 ***	-1 +/-0 ***	1 +/-6
0.25	0 +/-0 ***	0 +/-0 ***	0 +/-0 ***	0 +/-0 ***	0 +/-0 ***	-1 +/-0 ***	-2 +/-2
0.74	0 +/-0 ***	0 +/-0 ***	0 +/-0 ***	0 +/-0 ***	0 +/-0 ***	0 +/-2	1 +/-5
2.22	0 +/-0 ***	0 +/-0 ***	0 +/-0 ***	0 +/-0 ***	0 +/-0 ***	-1 +/-0 ***	7 +/-17
6.67	1 +/-2	1 +/-1	0 +/-0 ***	2 +/-4	2 +/-3	3 +/-6	5 +/-6
20	0 +/-8	2 +/-5	-1 +/-11	1 +/-6	0 +/-8	5 +/-8	8 +/-5 *

HSA synergy and antagonism
