# Supplementary material for: Therapeutic targeting of measles virus polymerase with ERDRP-0519 suppresses all RNA synthesis activity
Source: PLoS Pathog. 2021 Feb 23;17(2):e1009371. doi: 10.1371/journal.ppat.1009371 (PMC7935272; doi:10.1371/journal.ppat.1009371)
Supplement: S12 Fig — A) Linear schematic of the MeV L protein displaying areas surrounding the ERDRP-0519 binding pocket. B) Consensus sequences based on the complete L protein sequence alignments from S3 Fig complete L protein sequences in the NCBI virus database [67] for MeV (332 sequences), CDV (178 sequences), and PPRV (55 sequences, S10 Data) were generated with WebLogo [68]. The specific residues surrounding the predicted binding pocket are boxed. C) Spatial organization of different regions surrounding the ERDRP-0519 binding pocket. Segments are labeled by residue range. (PDF) [file ppat.1009371.s012.pdf]

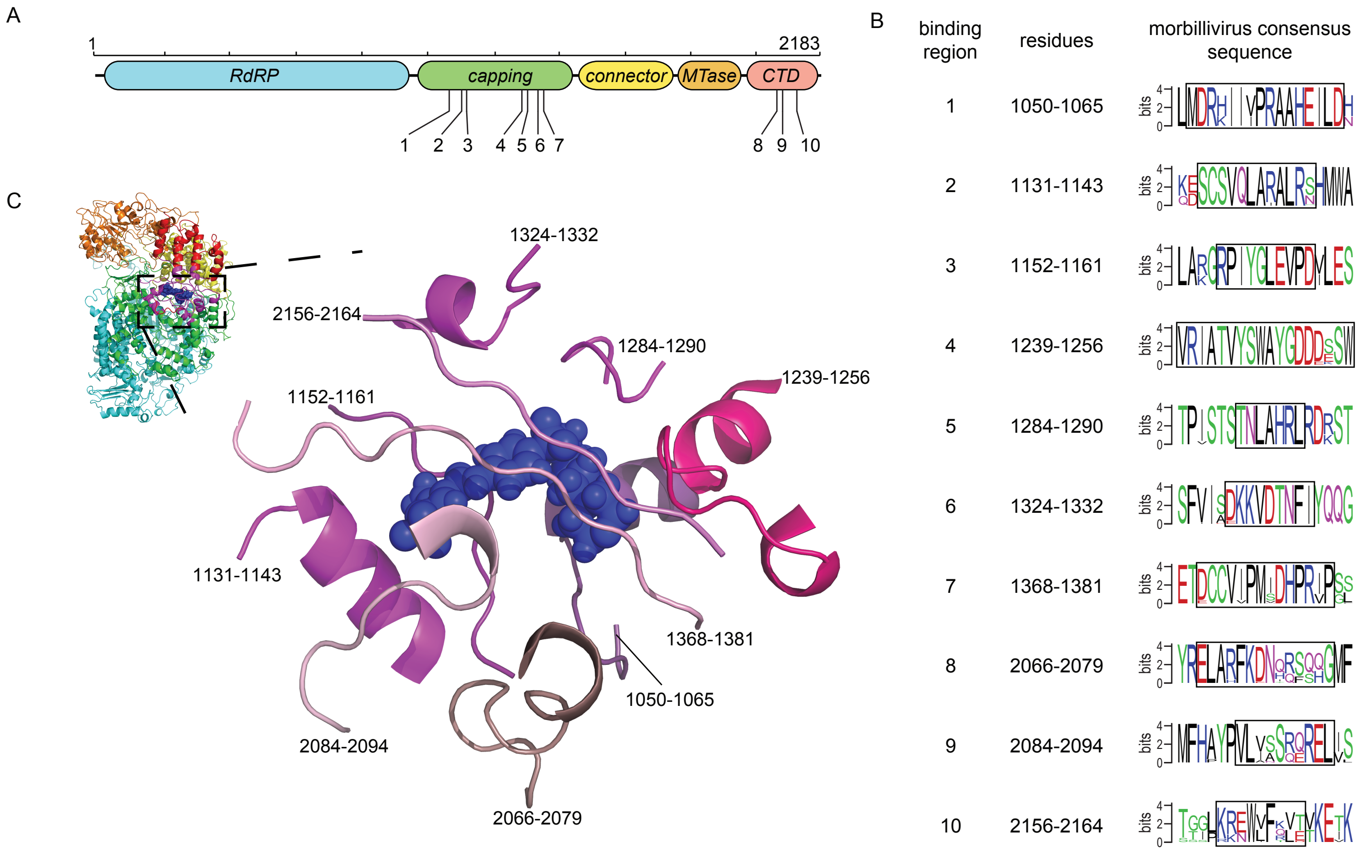
A
1
2183
RdRP
capping
connector
MTase
CTD
1 2 3 4 5 6 7 8 9 10
B
binding region
residues
morbillivirus consensus sequence
1 1050-1065
2 1131-1143
3 1152-1161
4 1239-1256
5 1284-1290
6 1324-1332
7 1368-1381
8 2066-2079
9 2084-2094
10 2156-2164
C
1324-1332
2156-2164
1284-1290
1239-1256
1152-1161
1131-1143
1368-1381
1050-1065
2084-2094
2066-2079
