# Supplementary material for: Therapeutic targeting of measles virus polymerase with ERDRP-0519 suppresses all RNA synthesis activity
Source: PLoS Pathog. 2021 Feb 23;17(2):e1009371. doi: 10.1371/journal.ppat.1009371 (PMC7935272; doi:10.1371/journal.ppat.1009371)
Supplement: S13 Fig — A-F) Docks of ERDRP-0519 into PIV-5 L (red sticks (A-B)), RSV L (red sticks (C-D)) and VSV L (red sticks (E-F)) did not yield poses similar to that obtained for docking into MeV L (orange sticks). 2-D schematics of the top scoring docking poses are shown for each structure. Peptide 1 (pink), peptide 2 (purple), the intrusion (blue) and priming (red) loops, ERDRP-0519 resistance mutations (black spheres), and the proposed PRNTase motifs (blue spheres) are marked. (PDF) [file ppat.1009371.s013.pdf]

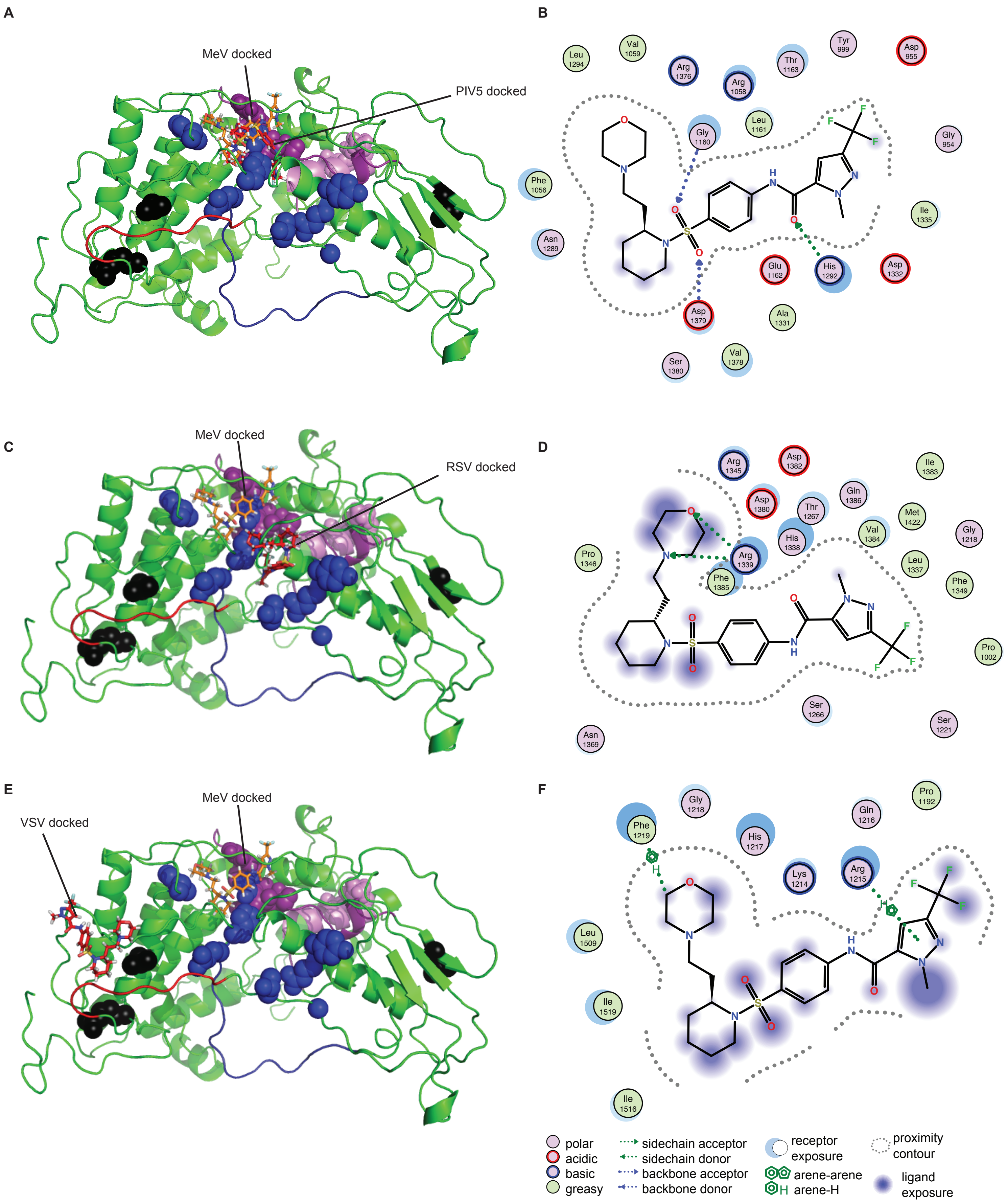

A
MeV docked
PIV5 docked
B
Leu 1294
Val 1059
Arg 1376
Arg 1058
Thr 1163
Tyr 999
Asp 955
Gly 1160
Leu 1161
Gly 954
Phe 1056
Ile 1335
Asn 1289
Glu 1162
His 1292
Asp 1332
Asp 1379
Ala 1331
Ser 1380
Val 1378
C
MeV docked
RSV docked
D
Arg 1345
Asp 1382
Ile 1383
Gln 1386
Asp 1380
Thr 1267
Met 1422
Gly 1218
Val 1384
His 1338
Pro 1346
Arg 1339
Leu 1337
Phe 1385
Phe 1349
Pro 1002
Ser 1266
Ser 1221
Asn 1369
E
VSV docked
MeV docked
F
Pro 1192
Gly 1218
Gln 1216
Phe 1219
His 1217
Lys 1214
Arg 1215
Leu 1509
Ile 1519
Ile 1516
polar
acidic
basic
greasy
sidechain acceptor
sidechain donor
backbone acceptor
backbone donor
receptor exposure
arene-arene
arene-H
proximity contour
ligand exposure
