# Supplementary material for: Therapeutic targeting of measles virus polymerase with ERDRP-0519 suppresses all RNA synthesis activity
Source: PLoS Pathog. 2021 Feb 23;17(2):e1009371. doi: 10.1371/journal.ppat.1009371 (PMC7935272; doi:10.1371/journal.ppat.1009371)
Supplement: S14 Fig — A-I) Overlay of the ERDRP-0519 docking pose into MeV L homology models based on PIV-5 (intrusion loop down; A, D, G), RABV (intrusion loop up; B, E, H), and RSV (intrusion loop up; C, F, I) L proteins. Close up views of the ERDRP-0519 binding pocket in the different MeV L models are shown in (D-F). Steric incompatibilities (red scaffold substructures) are noted with RABV and RSV-based models (H-I) with intrusion loop in initiation conformation. (PDF) [file ppat.1009371.s014.pdf]

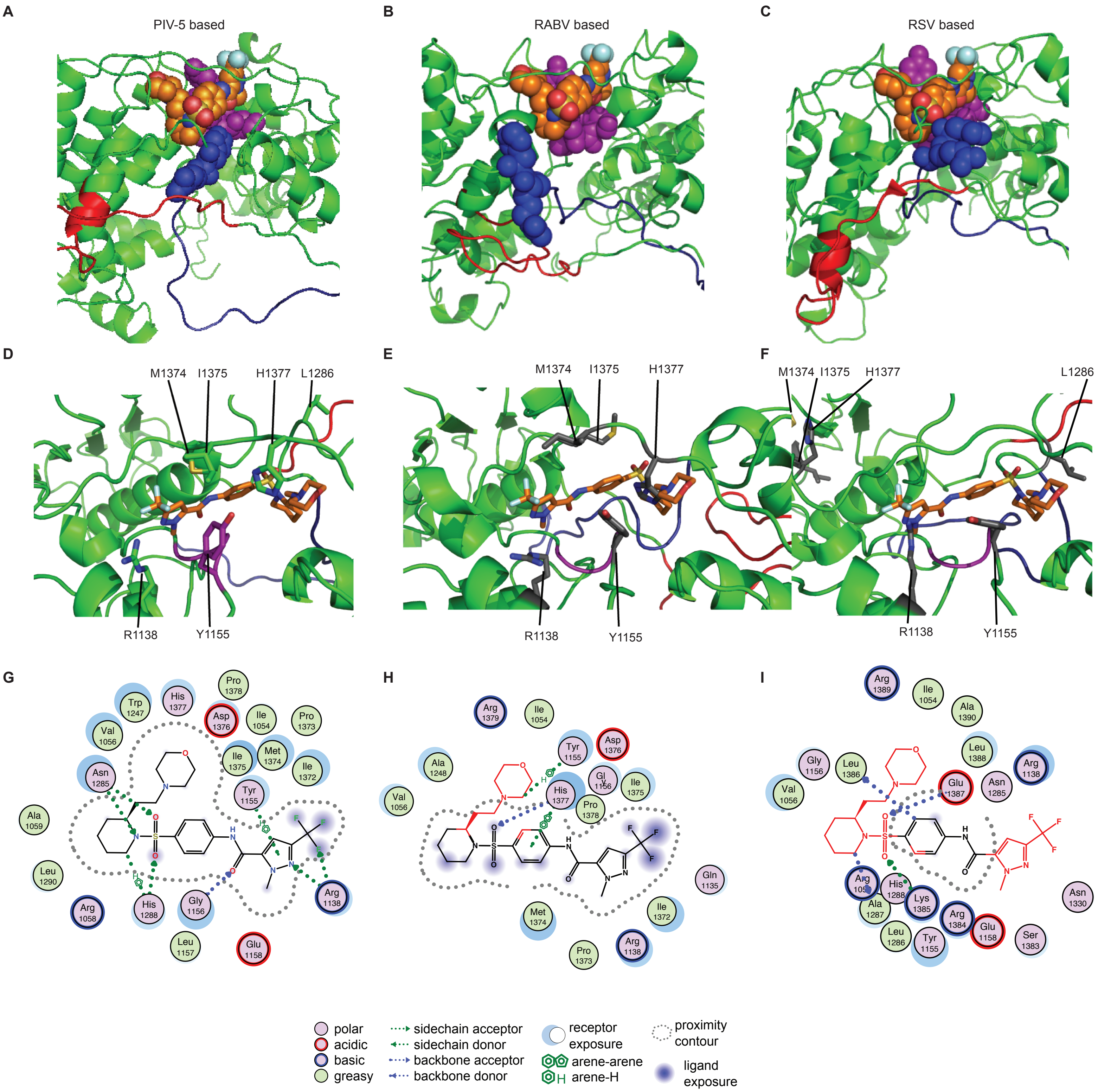
A
PIV-5 based
B
RABV based
C
RSV based
D
M1374
I1375
H1377
L1286
R1138
Y1155
E
M1374
I1375
H1377
R1138
Y1155
F
M1374
I1375
H1377
L1286
R1138
Y1155
G
H
I
polar
acidic
basic
greasy
sidechain acceptor
sidechain donor
backbone acceptor
backbone donor
receptor exposure
arene-arene
arene-H
proximity contour
ligand exposure
