# Supplementary material for: Therapeutic targeting of measles virus polymerase with ERDRP-0519 suppresses all RNA synthesis activity
Source: PLoS Pathog. 2021 Feb 23;17(2):e1009371. doi: 10.1371/journal.ppat.1009371 (PMC7935272; doi:10.1371/journal.ppat.1009371)
Supplement: S1 Table — (DOCX) [file ppat.1009371.s016.docx]

**Supporting table S1:** Mass spectrometry analysis of recombinant P-L complex preparations. Most abundant (iBAQ ≥10^9^) proteins identified by mass spectrometry are shown.

| **Majority protein IDs** | **Description** | **Mol. weight [kDa]** | **Sequence coverage [%]** | **Razor + unique peptides** | **Intensity** | **MS/MS Count** | **iBAQ^a^** |
| --- | --- | --- | --- | --- | --- | --- | --- |
| **MeV P** | **RdRP cofactor** | **56** | **95** | **78** | **7.81×10^11^** | **1715** | **3.12×10^10^** |
| **MeV L** | **RdRP** | **248** | **84** | **208** | **6.74×10^11^** | **1359** | **6.48×10^9^** |
| A0A2H1X3Z3 | mRNA cleavage factor complex* | 18 | 87 | 8 | 4.37×10^10^ | 87 | **1.09×10^10^** |
| A0A2H1WRK1 | mRNA cleavage factor complex* | 26 | 92 | 28 | 1.23×10^11^ | 285 | **1.02×10^10^** |
| A0A2H1VAM0 | mRNA cleavage factor complex* | 55 | 35 | 14 | 8.85×10^10^ | 96 | **5.53×10^9^** |
| A0A2H1VKI1 | Thioredoxin-like* | 47 | 66 | 36 | 1.04×10^11^ | 161 | **5.45×10^9^** |
| A0A2H1WKT6 | SCD6 protein-related, RNA metabolism* | 57 | 66 | 29 | 7.23×10^10^ | 211 | **4.52×10^9^** |
| A0A2H1WFA2 | Heat-Shock Protein | 72 | 87 | 56 | 1.14×10^11^ | 253 | **3.56×10^9^** |
| A0A2H1W5E0 | Scaffold protein* | 30 | 87 | 18 | 4.08×10^10^ | 131 | **2.72×10^9^** |
| A0A2H1VCM4 | Peroxiredoxin* | 40 | 43 | 18 | 6.25×10^10^ | 82 | **2.72×10^9^** |
| A0A2H1WKS6 | Ubiquitin-associated protein 2-like* | 63 | 73 | 17 | 2.62×10^10^ | 123 | **2.02×10^9^** |
| Q962T8 | 60S ribosomal protein L27a | 17 | 46 | 10 | 1.59×10^10^ | 25 | **1.98×10^9^** |
| A0A2H1VM87 | Scaffold protein with Ubiquitin-associated domain* | 41 | 51 | 15 | 2.43×10^10^ | 93 | **1.87×10^9^** |
| A0A2H1WQZ8 | 40S ribosomal protein S9* | 18 | 35 | 14 | 1.69×10^10^ | 22 | **1.69×10^9^** |
| A0A2H1WFG2 | Metal ion transmembrane transporter activity* | 38 | 58 | 14 | 9.32×10^9^ | 62 | **1.55×10^9^** |
| P17501;A0A097PUZ4;Q65336 | Baculovirus Major envelope glycoprotein | 59 | 52 | 27 | 3.52×10^10^ | 130 | **1.53×10^9^** |
| A0A2H1WX17;Q8I866 | Heat-Shock Protein | 73 | 75 | 43 | 4.56×10^10^ | 167 | **1.52×10^9^** |
| A0A2H1VC46 | Ataxin-2-like protein* | 67 | 64 | 29 | 3.74×10^10^ | 132 | **1.50×10^9^** |
| A0A2H1WWH2 | Protein yippee-like | 14 | 58 | 6 | 9.80×10^9^ | 18 | **1.40×10^9^** |
| A0A2H1VDL3 | Thioredoxin-like* | 24 | 78 | 11 | 1.62×10^10^ | 50 | **1.35×10^9^** |
| A0A2H1VXJ0 | LSM12-like protein* | 22 | 79 | 18 | 1.38×10^10^ | 51 | **1.25×10^9^** |
| S5G646;P41432 | Baculovirus late expression factor 6 | 20 | 67 | 13 | 1.21×10^10^ | 57 | **1.21×10^9^** |
| A0A2H1WJQ0 | L-lactate dehydrogenase* | 27 | 71 | 13 | 1.19×10^10^ | 39 | **1.19×10^9^** |
| A0A2H1VF70 | Nuclear Transport Factor 2-like | 25 | 78 | 14 | 1.17×10^10^ | 58 | **1.17×10^9^** |
| A0A2H1W9M2 | Scaffold protein* | 40 | 91 | 22 | 1.66×10^10^ | 82 | **1.11×10^9^** |
| A0A2H1W5D9 | Scaffold protein* | 34 | 29 | 7 | 1.71×10^10^ | 30 | **1.00×10^9^** |
| A0A2H1VWC5 | Lupus La protein-like* | 84 | 69 | 50 | 4.31×10^10^ | 136 | **1.00×10^9^** |
| *^a^*: Intensity Based Absolute Quantification | | | |  |  |  |  |
| *: predicted by molecular domain or sequence homology | |  |  |  |  |  |  |
