# Supplementary material for: Therapeutic targeting of measles virus polymerase with ERDRP-0519 suppresses all RNA synthesis activity
Source: PLoS Pathog. 2021 Feb 23;17(2):e1009371. doi: 10.1371/journal.ppat.1009371 (PMC7935272; doi:10.1371/journal.ppat.1009371)
Supplement: S2 Table — (DOCX) [file ppat.1009371.s017.docx]

**Supporting table S2:** ERDRP-0519 analogs used for development of the 3D-QSAR model. Experimental [11, 41, 64] and predicted activities are shown.

| Name | Set | SMILES | EC_50_  [µM] | CC_50_  [µM] | predicted pIC_50_ | pIC_50_ |
| --- | --- | --- | --- | --- | --- | --- |
| 15a | training | S(=O)(=O)(N1CCCCC1)c1ccc(NC(=O)c2n(C)cc(C(F)(F)F)c2)cc1 | 0.014 | >300 | 8.808 | 7.853 |
| 15b | training | S(=O)(=O)(N1C(C)CCCC1)c1ccc(NC(=O)c2n(C)cc(C(F)(F)F)c2)cc1 | 0.028 | >300 | 7.409 | 7.537 |
| 15c | training | S(=O)(=O)(N1CCC(C)CC1)c1ccc(NC(=O)c2n(C)cc(C(F)(F)F)c2)cc1 | 0.035 | >300 | 7.947 | 7.455 |
| 15d | training | S(=O)(=O)(N1C(C)CCCC1C)c1ccc(NC(=O)c2n(C)cc(C(F)(F)F)c2)cc1 | 0.014 | >300 | 7.755 | 7.853 |
| 15e | training | S(=O)(=O)(N1C(CC)CCCC1)c1ccc(NC(=O)c2n(C)cc(C(F)(F)F)c2)cc1 | 0.086 | >300 | 7.342 | 7.060 |
| 15f | training | S(=O)(=O)(N1CCCCCC1)c1ccc(NC(=O)c2n(C)cc(C(F)(F)F)c2)cc1 | 0.005 | >300 | 7.768 | 8.301 |
| 15g | training | S(=O)(=O)(N1CCCCCCC1)c1ccc(NC(=O)c2n(C)cc(C(F)(F)F)c2)cc1 | 0.045 | >300 | 7.609 | 7.346 |
| 15h | training | S(=O)(=O)(N1[C@@H](C(=O)OC(=O)c2ccccc2)CCC1)c1ccc(NC(=O)c2n(C)cc(C(F)(F)F)c2)cc1 | 14 | >100 | 4.876 | 4.853 |
| 15i | training | S(=O)(=O)(N1C(C(=O)O)CCC1)c1ccc(NC(=O)c2n(C)cc(C(F)(F)F)c2)cc1 | 23 | >300 | 5.569 | 4.638 |
| 15k | training | S(=O)(=O)(N1CCC(C(F)(F)F)CC1)c1ccc(NC(=O)c2n(C)cc(C(F)(F)F)c2)cc1 | 0.02 | 159 | 7.670 | 7.698 |
| 15m | training | S(=O)(=O)(N(C)C)c1ccc(NC(=O)c2n(C)cc(C(F)(F)F)c2)cc1 | 6.3 | >300 | 5.319 | 5.200 |
| 15n | training | S(=O)(=O)(N(CC)CC)c1ccc(NC(=O)c2n(C)cc(C(F)(F)F)c2)cc1 | 0.018 | >300 | 7.751 | 7.721 |
| 15o | training | S(=O)(=O)(N(C(C)C)C(C)C)c1ccc(NC(=O)c2n(C)cc(C(F)(F)F)c2)cc1 | 0.52 | >300 | 6.059 | 6.275 |
| 15p | training | S(=O)(=O)(N(CCC)CCC)c1ccc(NC(=O)c2n(C)cc(C(F)(F)F)c2)cc1 | 0.19 | >300 | 6.677 | 6.721 |
| 15r | training | S(=O)(=O)(N(CC=C)CC=C)c1ccc(NC(=O)c2n(C)cc(C(F)(F)F)c2)cc1 | 3.3 | 34 | 5.509 | 5.481 |
| 16677 | training | S(=O)(=O)(N1CCCC1)c1ccc(NC(=O)c2n(C)nc(C(F)(F)F)c2)cc1 | 0.23 | >300 | 6.691 | 6.619 |
| 1a | test | S(=O)(=O)(N1C(COCOC)CCCC1)c1ccc(NC(=O)c2n(C)nc(C(F)(F)F)c2)cc1 | 1.5 | >100 | 7.273 | 5.823 |
| 28c | test | S(=O)(=O)(N1C(CO)CCCC1)c1ccc(NC(=O)c2n(C)nc(C(F)(F)F)c2)cc1 | 0.85 | 159 | 7.903 | 6.070 |
| 2g | test | S(=O)(=O)(N1C(C=CC(=O)OCC)CCCC1)c1ccc(NC(=O)c2n(C)nc(C(F)(F)F)c2)cc1 | 6.8 | >100 | 4.478 | 5.167 |
| 2i | test | S(=O)(=O)(N1C(CCC(=O)OCC)CCCC1)c1ccc(NC(=O)c2n(C)nc(C(F)(F)F)c2)cc1 | 6.6 | >100 | 5.488 | 5.173 |
| 2k | test | S(=O)(=O)(N1[C@H](CCO)CCCC1)c1ccc(NC(=O)c2n(C)nc(C(F)(F)F)c2)cc1 | 0.1 | >100 | 8.330 | 7.00 |
| 3a | test | S(=O)(=O)(c1ccc(NC(=O)c2n(C)nc(C(F)(F)F)c2)cc1)c1ccccc1 | 0.09 | >100 | 7.253 | 7.045 |
| 3b | test | S(=O)(=O)(c1c(OC)cccc1)c1ccc(NC(=O)c2n(C)nc(C(F)(F)F)c2)cc1 | 3.0 | >100 | 6.062 | 5.508 |
| 3c | test | S(=O)(=O)(c1c(O)cccc1)c1ccc(NC(=O)c2n(C)nc(C(F)(F)F)c2)cc1 | 4.5 | 75 | 6.851 | 5.346 |
| 3d | test | S(=O)(=O)(c1c(OC(=O)C)cccc1)c1ccc(NC(=O)c2n(C)nc(C(F)(F)F)c2)cc1 | 4.5 | >100 | 4.886 | 5.346 |
| 9a | test | S(=O)(=O)(N1C(C)CCCC1)c1ccc(NC(=O)c2n(CC)nc(C(F)(F)F)c2)cc1 | 0.055 | 30 | 8.253 | 7.259 |
| AS-105 | test | S(=O)(=O)(N1[C@@H](C(=O)O)CCC1)c1ccc(NC(=O)c2n(C)nc(C(F)(F)F)c2)cc1 | 23 | >300 | 4.616 | 4.638 |
| AS-136a | test | S(=O)(=O)(N1CCCCC1)c1ccc(NC(=O)c2n(C)nc(C(F)(F)F)c2)cc1 | 0.014 | >300 | 7.614 | 7.853 |
| ERDRP-0519 | test | S(=O)(=O)(N1[C@H](CCN2CCOCC2)CCCC1)c1ccc(NC(=O)c2n(C)nc(C(F)(F)F)c2)cc1 | 0.059 | 75 | 8.083 | 7.221 |
| ERDRP-0519_az_ | test | S(=O)(=O)(N1[C@@H](CCOCc2ccc(N=[N+]=[N-])cc2)CCCC1)c1ccc(NC(=O)c2n(C)nc(C(F)(F)F)c2)cc1 | 12.1 | >100 | 4.920 | 5.572 |
| MS-12 | test | S(=O)(=O)(N1CCCC1)c1ccc(NC(=O)c2n(-c3c(C(F)(F)F)cccc3)ccn2)cc1 | 65 | >300 | 5.385 | 4.187 |
| MS-26 | test | S(=O)(=O)(N1CCCC1)c1ccc(CC(=O)c2n(C)nc(C(F)(F)F)c2)cc1 | 15.5 | >300 | 6.048 | 4.809 |
| MS-27 | test | S(=O)(=O)(N1CCCC1)c1ccc(CC(O)c2n(C)nc(C(F)(F)F)c2)cc1 | 41 | >300 | 5.866 | 4.387 |
