# Supplementary material for: Therapeutic targeting of measles virus polymerase with ERDRP-0519 suppresses all RNA synthesis activity
Source: PLoS Pathog. 2021 Feb 23;17(2):e1009371. doi: 10.1371/journal.ppat.1009371 (PMC7935272; doi:10.1371/journal.ppat.1009371)

S4 Dataset. Source and biological repeats from figure 3D.

Autoradiogram of *primer extension* RdRP assay with **MeV L H589Y** after fractionation through Urea-PAGE

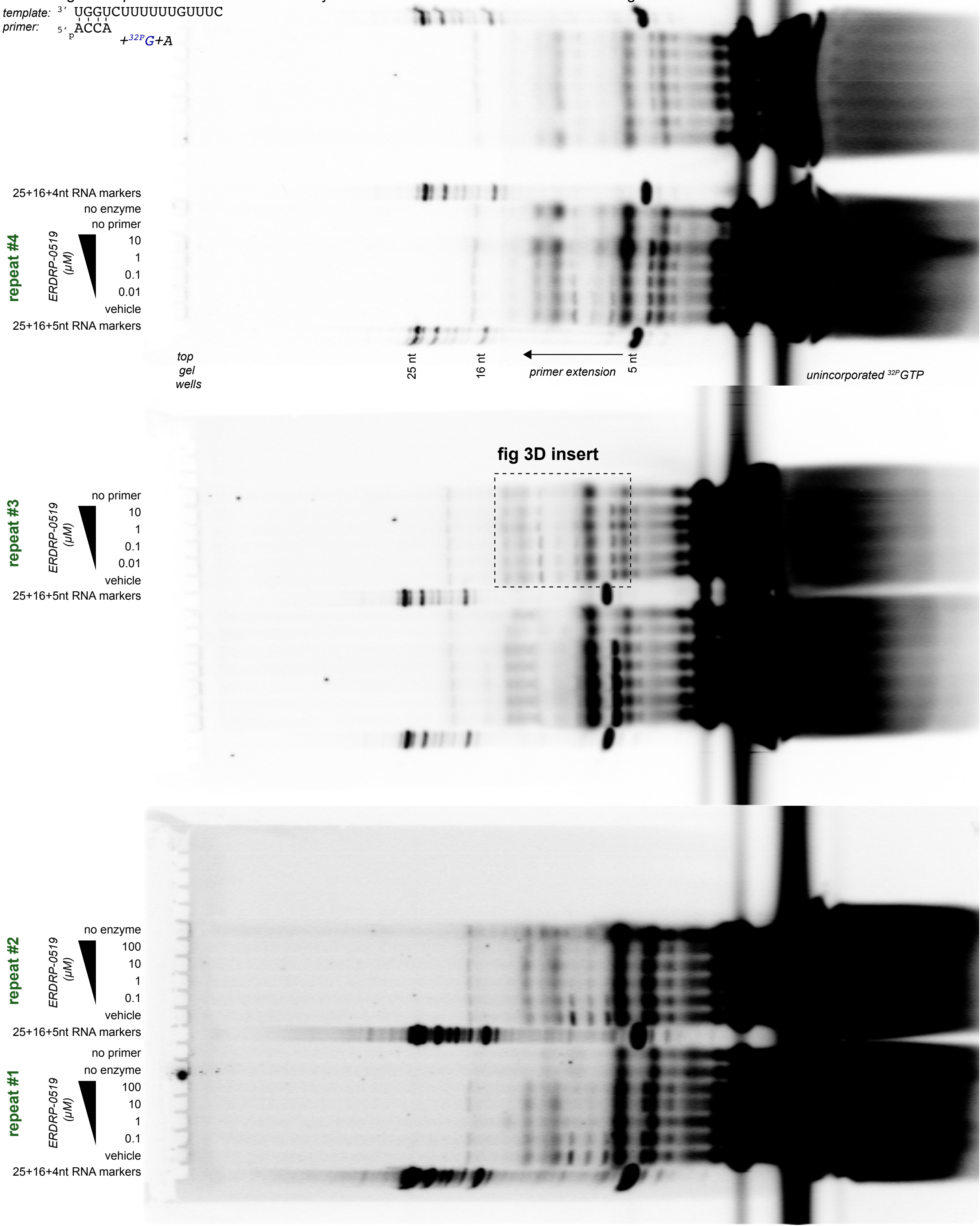

S4 Dataset. Source and biological repeats from figure 3D.

Autoradiogram of *primer extension* RdRP assay with **MeV L T776A** after fractionation through Urea-PAGE

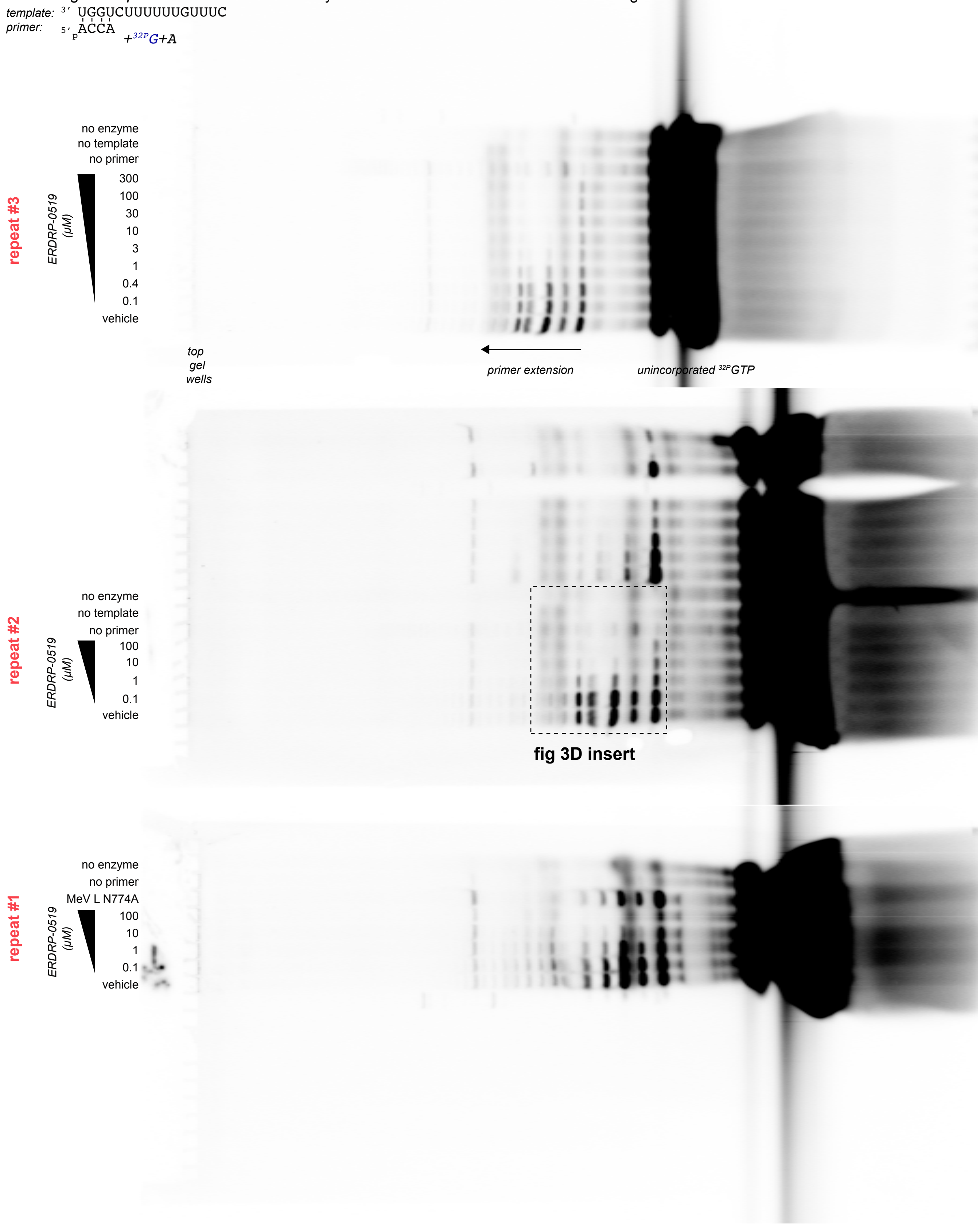

S4 Dataset. Source and biological repeats from figure 3D.

Autoradiogram of *primer extension* RdRP assay with **MeV L T776A** after fractionation through Urea-PAGE

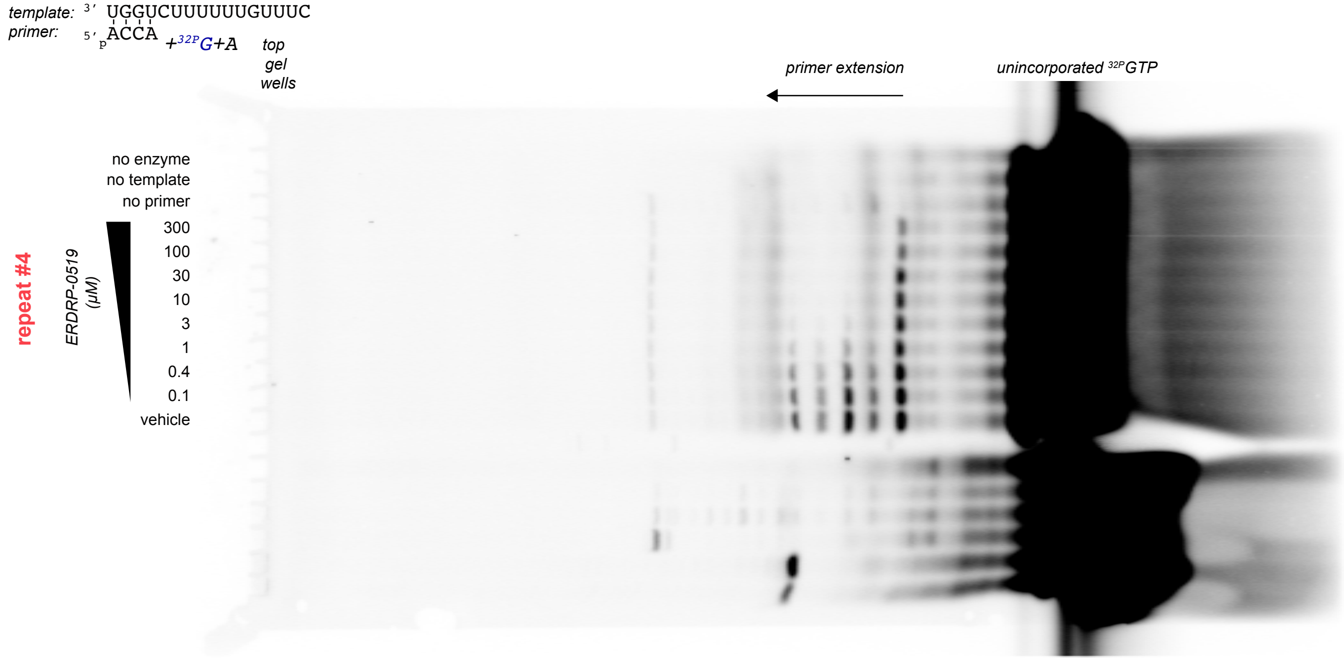

S4 Dataset. Source and biological repeats from figure 3D.

Autoradiogram of *primer extension* RdRP assay with **MeV L WT** after fractionation through Urea-PAGE

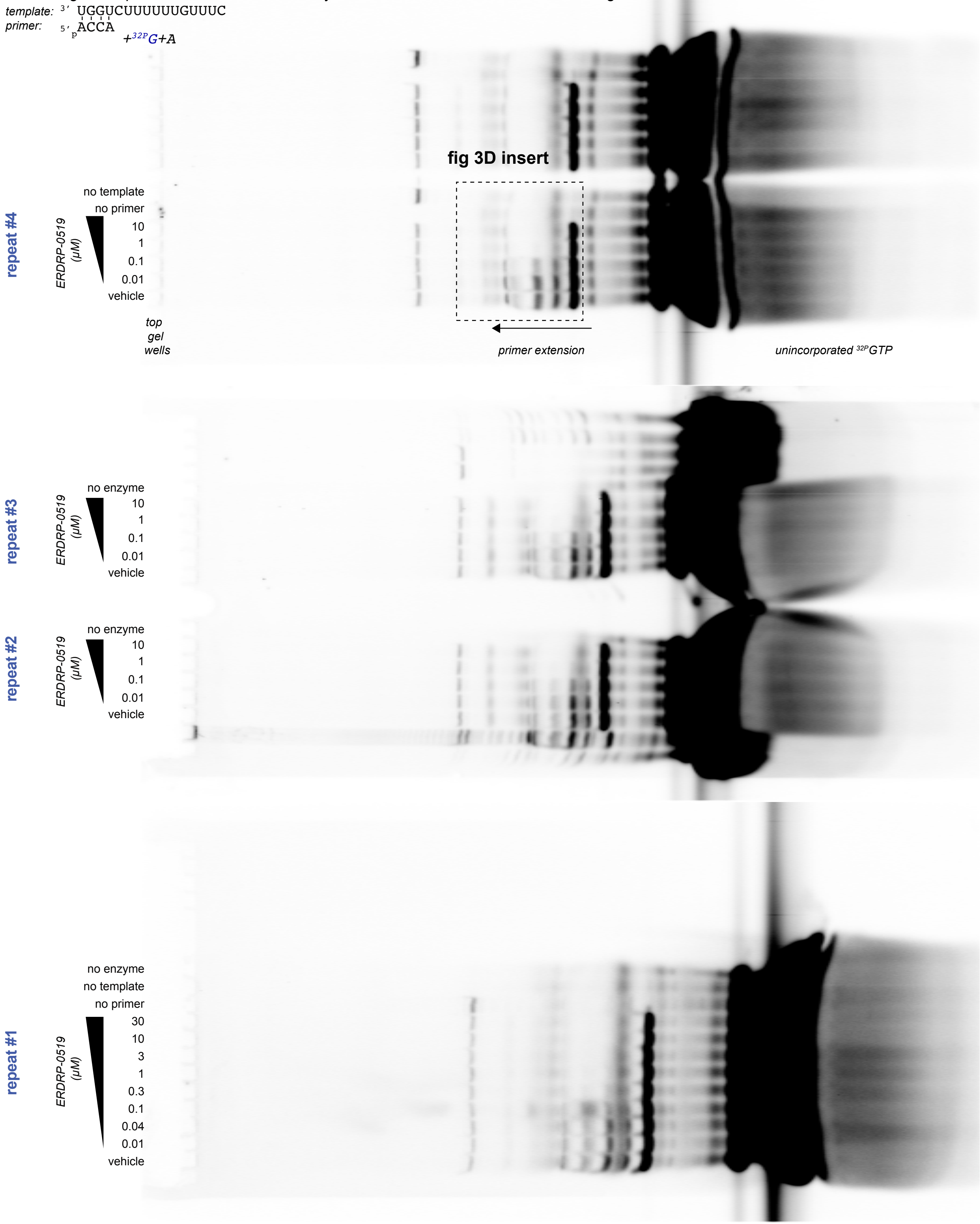

Supplement: S4 Data — (PDF) [file ppat.1009371.s021.pdf]
