# Supplementary material for: Therapeutic targeting of measles virus polymerase with ERDRP-0519 suppresses all RNA synthesis activity
Source: PLoS Pathog. 2021 Feb 23;17(2):e1009371. doi: 10.1371/journal.ppat.1009371 (PMC7935272; doi:10.1371/journal.ppat.1009371)

S5 Dataset. Source and biological repeats from supporting figure S8.

Autoradiogram of *primer extension* RdRP assay with MeV L RdRP catalytic mutants after fractionation through Urea-PAGE

template: 3′ UGGUCUUUUUUGUUUC

primer: 5′ pACCA

+^{32P}*G*+*A*

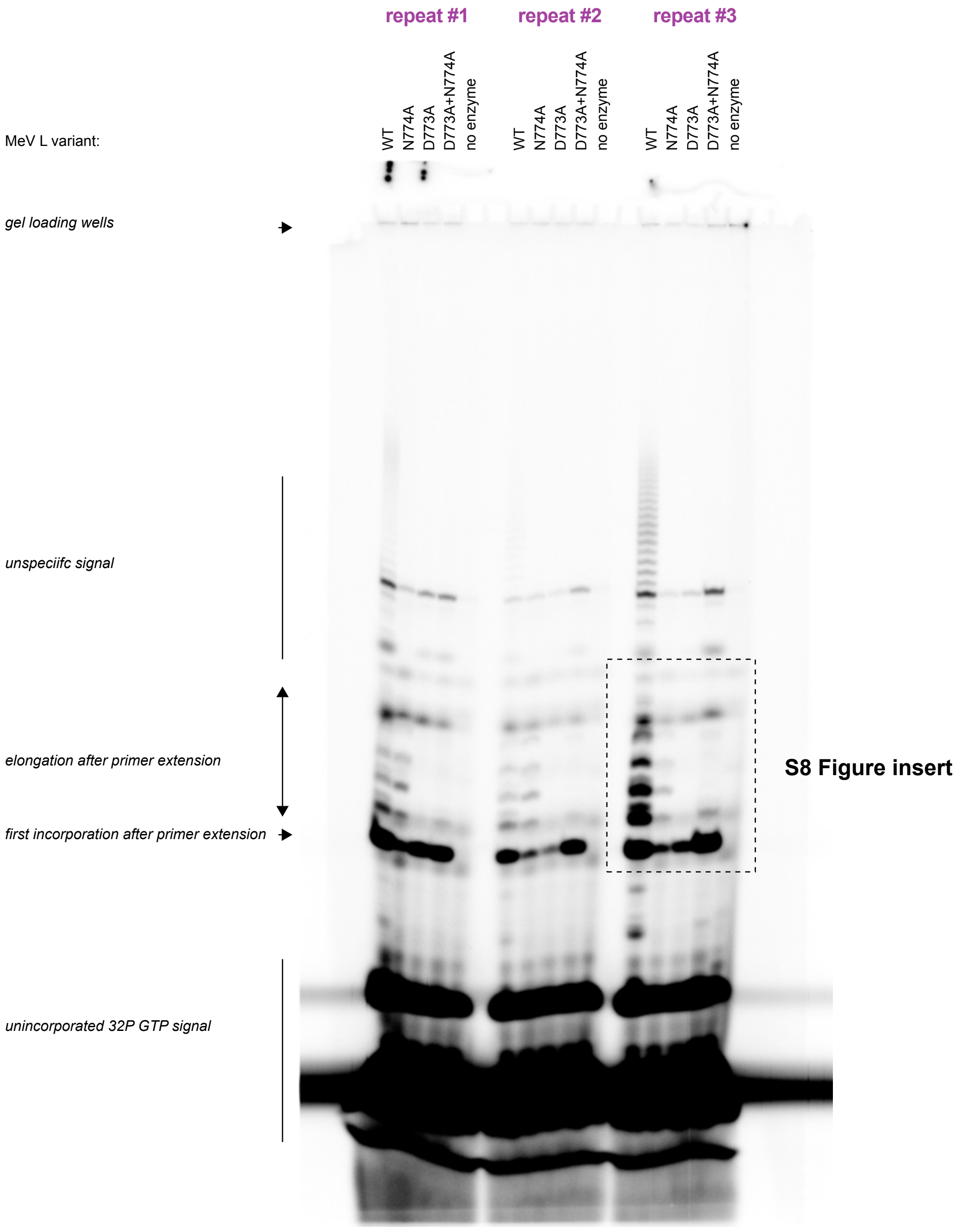

Supplement: S5 Data — (PDF) [file ppat.1009371.s022.pdf]
