# Supplementary material for: Therapeutic targeting of measles virus polymerase with ERDRP-0519 suppresses all RNA synthesis activity
Source: PLoS Pathog. 2021 Feb 23;17(2):e1009371. doi: 10.1371/journal.ppat.1009371 (PMC7935272; doi:10.1371/journal.ppat.1009371)

S6 Dataset. Source and biological repeats from figure 4B-C.

Autoradiogram of *de novo* RdRP assay with **MeV L WT** after fractionation through Urea-PAGE

template:

3′ UGCUCUUUUUUUCACAGUUUUUGAU

+^{32P}G+A+U+C

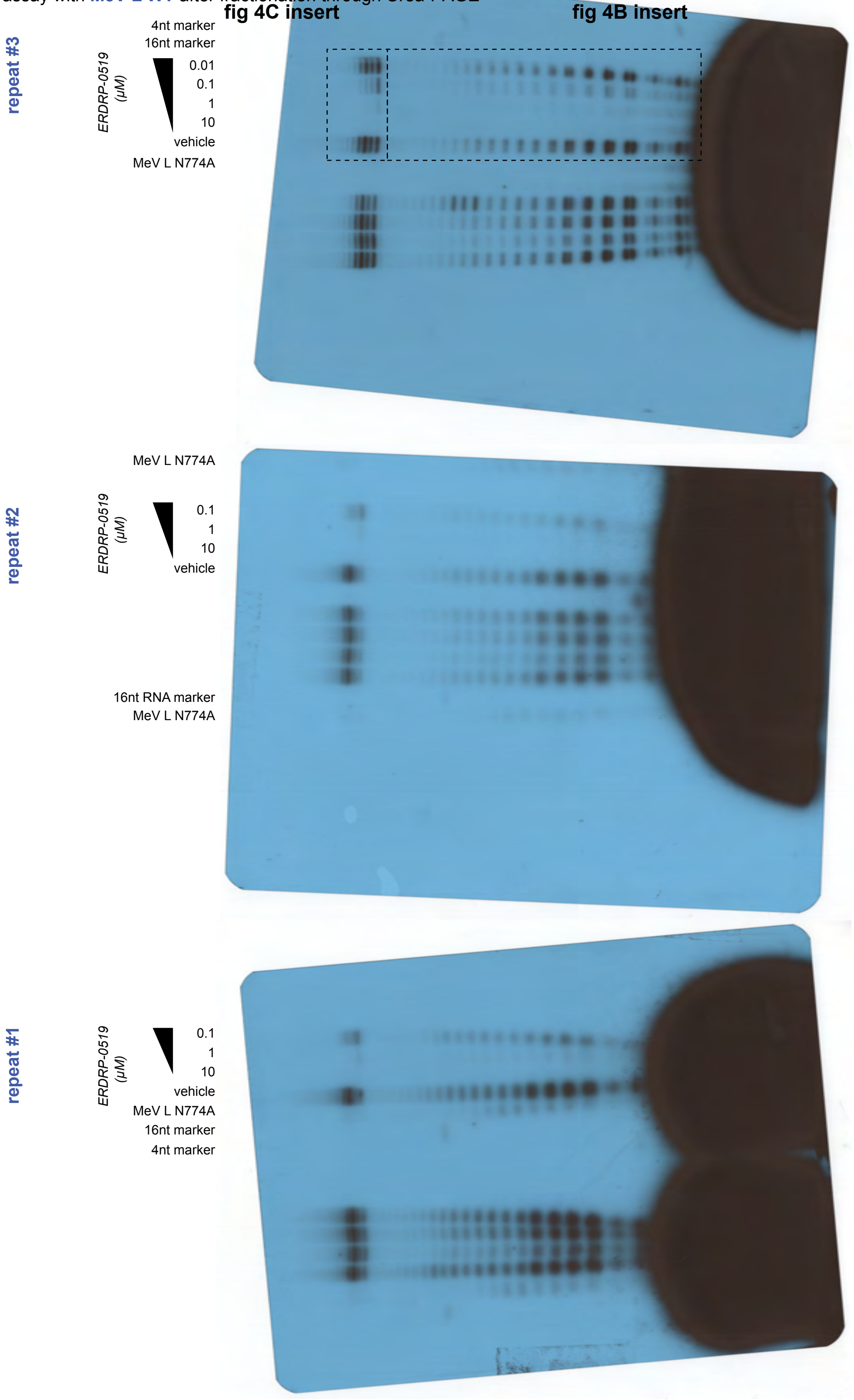

Supplement: S6 Data — (PDF) [file ppat.1009371.s023.pdf]
