# Supplementary material for: Therapeutic targeting of measles virus polymerase with ERDRP-0519 suppresses all RNA synthesis activity
Source: PLoS Pathog. 2021 Feb 23;17(2):e1009371. doi: 10.1371/journal.ppat.1009371 (PMC7935272; doi:10.1371/journal.ppat.1009371)

S8 Dataset. Source and biological repeats from supporting figure S9.

Autoradiogram of *primer extension* RdRP assay with MeV L and ERDRP-0519az after fractionation through Urea-PAGE

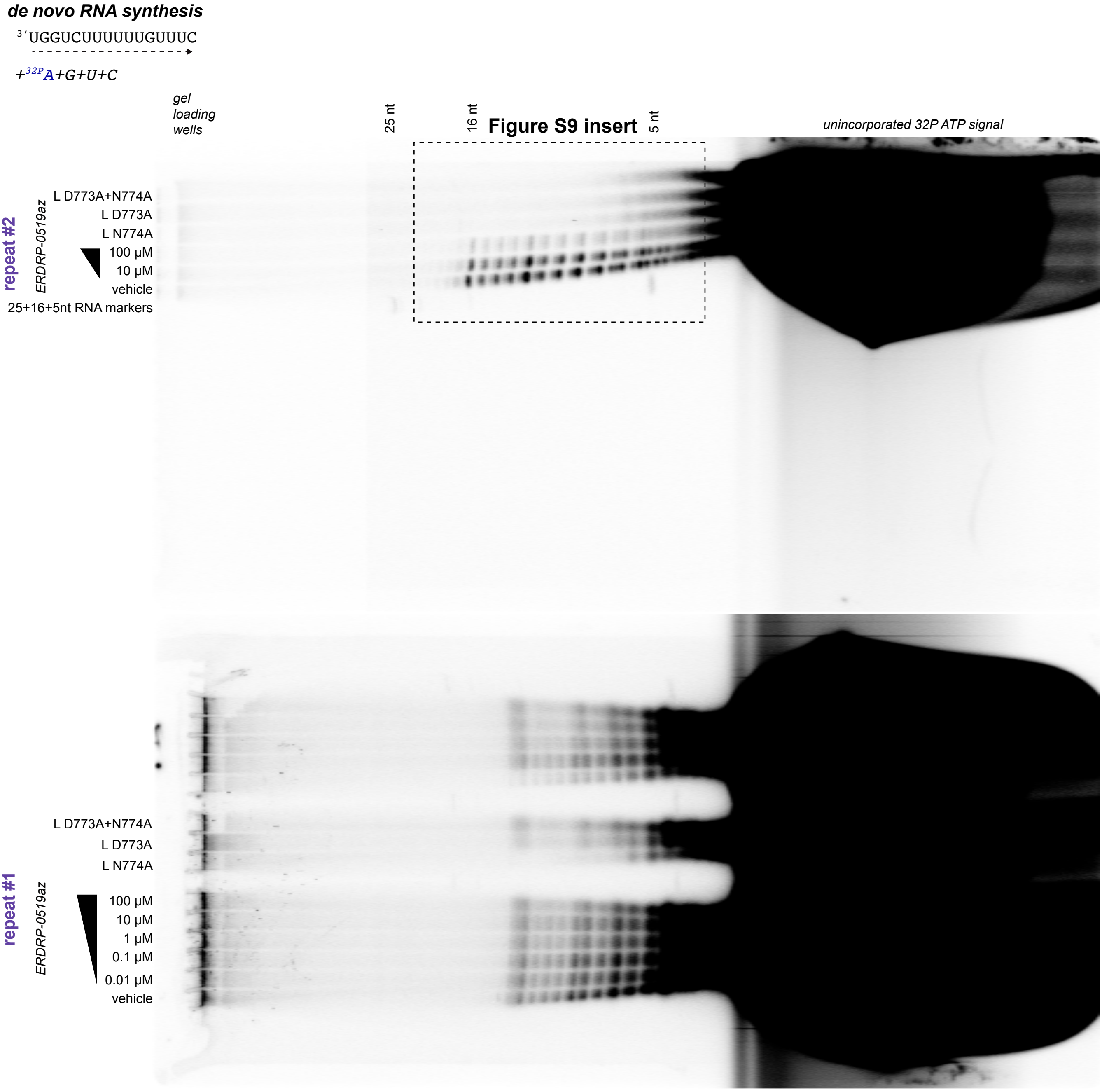

Supplement: S8 Data — (PDF) [file ppat.1009371.s025.pdf]
