# Supplementary material for: Therapeutic targeting of measles virus polymerase with ERDRP-0519 suppresses all RNA synthesis activity
Source: PLoS Pathog. 2021 Feb 23;17(2):e1009371. doi: 10.1371/journal.ppat.1009371 (PMC7935272; doi:10.1371/journal.ppat.1009371)
Supplement: S10 Data — (DOCX) [file ppat.1009371.s027.docx]

| MeV L sequence | | | | CDV L sequences | | | PPRV L sequences |
| --- | --- | --- | --- | --- | --- | --- | --- |
| NP_056924.1 | QGN67835.1 | AVA07220.1 | AEP40437.1 | NP_047207.1 | AXE15491.1 | AAT94554.1 | AIN40493.1 |
| QOT47606.1 | QGN67843.1 | BBB34989.1 | AEP40445.1 | QOZ05857.1 | AXE15498.1 | AAS48413.1 | AIL54021.1 |
| QOP74987.1 | QGQ32885.1 | ASU91871.1 | AEP40453.1 | QOZ05864.1 | AXE15505.1 | AAR32274.1 | AIL53997.1 |
| QMU24335.1 | QGX04278.1 | AQT38243.1 | AEP40461.1 | QOZ05871.1 | AXE15512.1 | AAR30104.1 | AIL54029.1 |
| QMU24343.1 | QGW61774.1 | ARL00303.1 | AEP40469.1 | QOZ05878.1 | AXE15519.1 | AAR16541.1 | AIL54037.1 |
| QMU24351.1 | QGW61782.1 | ARL00311.1 | AEP40477.1 | QOZ05885.1 | AWX68682.1 | AAQ96301.1 | AJE30398.1 |
| QMU24359.1 | QGW61790.1 | ARL00319.1 | AEF30345.1 | QOZ05892.1 | AWX68697.1 | AAQ96309.1 | ARP51876.1 |
| QMU24367.1 | QGW61798.1 | ARL00327.1 | AEF30346.1 | QOZ05899.1 | BBC15217.1 | AAK54670.1 | AJA39815.1 |
| QMU24375.1 | QGW61806.1 | ARL00335.1 | AEF30347.1 | QOZ05906.1 | AWM96406.1 | AAG30921.1 | AJE30414.1 |
| QMU24383.1 | QGW61814.1 | ARL00343.1 | AEH42048.1 | QOZ05913.1 | AWM96416.1 | AAD49704.1 | ALM55671.1 |
| QMU24391.1 | QGW61822.1 | APM88381.1 | AEH42054.1 | QOZ05920.1 | AVD50303.1 | AAC26996.1 | AJE30405.1 |
| QMU24399.1 | QGW61830.1 | AOR52441.1 | BAK08876.1 | QOZ05927.1 | BBB35232.1 | CAA70842.1 | ALA65399.1 |
| QMU24407.1 | QGW61838.1 | AIJ02766.1 | BAJ51787.1 | QOZ05934.1 | ARS02978.1 | P24658.3 | AKN58854.1 |
| QMU24415.1 | QGW61846.1 | AIJ02774.1 | ADO17332.1 | QOZ05948.1 | ARQ16407.1 |  | AOO35468.1 |
| QMU24423.1 | QBC83009.1 | AIJ02782.1 | Q9WMB3.1 | QOZ05955.1 | ARQ80414.1 |  | ASN63850.1 |
| QMU24431.1 | QBC83017.1 | AIJ02790.1 | ACU57144.1 | QOZ05961.1 | ARQ80420.1 |  | ASN63856.1 |
| QMU24439.1 | QBC83025.1 | AIJ02798.1 | ACU57152.1 | QOZ05968.1 | ARQ80426.1 |  | ASN63862.1 |
| QMU24447.1 | QBC83033.1 | AIJ02806.1 | BAH96586.1 | QOZ05975.1 | ARQ80432.1 |  | ASN63868.1 |
| QMU24455.1 | QBC83041.1 | AIJ02814.1 | BAH96594.1 | QOZ05982.1 | ARQ80433.1 |  | AIK97760.1 |
| QMU24463.1 | QBC83049.1 | AIJ02822.1 | ACN54408.1 | QOZ05989.1 | ARI45564.1 |  | ANG60362.1 |
| QMU24471.1 | QBC83057.1 | AIJ02830.1 | ACN54416.1 | QOZ05996.1 | AMH87499.1 |  | AAS68032.2 |
| QMU24479.1 | QBC83065.1 | AMM63608.1 | ACN50015.1 | QKV43687.1 | AOV62790.1 |  | QGP72524.1 |
| QMU24487.1 | QBC83073.1 | AMM63616.1 | ACN50054.1 | QKV27502.1 | AOV62796.1 |  | QGN18695.1 |
| QMU24495.1 | QBC83081.1 | ALE27088.1 | ACN50062.1 | QKS75097.1 | AOV62802.1 |  | AGG09147.1 |
| QMU24503.1 | QBC83089.1 | ALE27094.1 | CAV33221.1 | QKS75106.1 | AOV62808.1 |  | AIL54005.1 |
| QMU24511.1 | QBC83097.1 | ALE27100.1 | CAV22257.1 | QJF12088.1 | AOV62814.1 |  | AHF58489.2 |
| QMU24519.1 | QBC83105.1 | ALE27106.1 | ACJ66777.1 | QJF12095.1 | BAW32699.1 |  | ART91290.1 |
| QMU24527.1 | QBC83113.1 | ALE27112.1 | ABU49611.1 | QJF12102.1 | APD29238.1 |  | YP_133828.1 |
| QMU24535.1 | QBC83121.1 | ALE27118.1 | ACA09727.1 | QJF12109.1 | AOO35462.1 |  | CAH61259.1 |
| QMU24543.1 | QBC83129.1 | ALE27124.1 | ABY21184.1 | QJF12116.1 | AOA33118.1 |  | AFR66767.1 |
| QMU24551.1 | QBC83137.1 | ALE27130.1 | ABY21192.1 | QJF12123.1 | ANG57137.1 |  | ABY71272.1 |
| QMU24559.1 | QBC83145.1 | ALE27136.1 | ABY21200.1 | QJF12130.1 | ANG57145.1 |  | ASN63874.1 |
| QMU24567.1 | QBC83153.1 | ALE27142.1 | ABY21208.1 | QJF12137.1 | AJW59282.1 |  | AEH25645.1 |
| QMU24575.1 | QBC83161.1 | ALE27148.1 | ABY21216.1 | QJF12144.1 | AJO72805.1 |  | ACQ44672.1 |
| QMU24583.1 | QBC83169.1 | ALE27154.1 | ABK40531.1 | QJF12151.1 | AJO72812.1 |  | AMX28328.1 |
| QMU24591.1 | QBC83177.1 | ALE27160.1 | BAE98300.1 | QJF12158.1 | AKP49171.1 |  | AMX28312.1 |
| QMU24599.1 | QBC83185.1 | ALE27166.1 | ABD34002.1 | QJF12165.1 | AKM12415.1 |  | AKR81282.1 |
| QMU24607.1 | QBC83193.1 | ALE27172.1 | ABD34010.1 | QJF12172.1 | AJP31604.1 |  | AKQ09545.1 |
| QMU24615.1 | QBC83201.1 | ALE27178.1 | ABD34018.1 | QJF12179.1 | AJP31611.1 |  | AMX28320.1 |
| QMU24623.1 | QBC83209.1 | ALE27184.1 | ABB71647.1 | QJF12186.1 | AJH76968.1 |  | ANS59484.1 |
| QMU24631.1 | QBC83217.1 | ALE27190.1 | ABB71655.1 | QJF12193.1 | AIM39349.1 |  | AKG94170.1 |
| QMU24639.1 | QBC83225.1 | ALE27196.1 | ABB71663.1 | QJF12207.1 | AIM39329.1 |  | AIL54013.1 |
| QMU24647.1 | QBC83233.1 | ALE27202.1 | ABB71671.1 | QJF12214.1 | AIN44018.1 |  | AJT59442.1 |
| QMU24655.1 | QBC83241.1 | ALE27208.1 | ABA59563.1 | QJF12228.1 | AIL92336.1 |  | ABX75305.1 |
| QMU24663.1 | AZK35994.1 | ALE27214.1 | AAV84959.1 | QJF12271.1 | AIK01744.1 |  | ANG60370.1 |
| QMU24671.1 | AZK36002.1 | ALE27220.1 | AAR32659.1 | QIX02893.1 | AHY03301.1 |  | AKT04308.1 |
| QMU24679.1 | ATD86884.1 | ALE27226.1 | AAR32667.1 | QBA86045.1 | AHN60084.1 |  | ANS54234.1 |
| QMU24687.1 | ATD86890.1 | ALE27232.1 | AAM89253.1 | QGN01268.1 | AHM26182.1 |  | AKT04324.1 |
| QMU24695.1 | ATD86896.1 | ALE27238.1 | AAM46767.1 | QCY53853.1 | AHF81427.1 |  | AID07003.1 |
| QMU24703.1 | AYU88786.1 | ALE27244.1 | BAB60955.1 | QCY53859.1 | BAO37343.1 |  | AIK19905.1 |
| QMU24711.1 | AYU88794.1 | ALE27250.1 | BAB60867.1 | QCY53865.1 | BAO37350.1 |  | ABX75313.1 |
| QMU24719.1 | AYU88802.1 | ALE27256.1 | AAF85666.1 | QCY53871.1 | BAO37357.1 |  | AUP34041.1 |
| QMU24727.1 | AXI82329.1 | ALE27262.1 | AAF85674.1 | QCY53877.1 | BAO37364.1 |  | ADX95996.1 |
| QMU24735.1 | AXI82336.1 | ALE27268.1 | AAF85682.1 | QCY53883.1 | BAO37371.1 |  | CAJ01701.1 |
| QMU24743.1 | AXI82343.1 | ALE27274.1 | AAF85690.1 | QCY53889.1 | BAO37378.1 |  | AKT04316.1 |
| QMU24751.1 | AXI82350.1 | ALE27280.1 | AAF85698.1 | QCY53895.1 | BAO37385.1 |  |  |
| QMU24759.1 | AXI82357.1 | ALE27286.1 | AAF85706.1 | QCY53901.1 | BAO37392.1 |  |  |
| QMU24767.1 | AXI82364.1 | ALE27292.1 | BAA84132.1 | QCY53907.1 | BAO03783.1 |  |  |
| QMU24775.1 | AXI82371.1 | ALE27298.1 | AAD29091.2 | QCY53913.1 | BAO03791.1 |  |  |
| QMU24783.1 | AXI82378.1 | ALE27304.1 | AAD29092.1 | QFE96894.1 | BAN16482.1 |  |  |
| QMU24791.1 | AXI82385.1 | ALE27310.1 | AAD29093.1 | QFE96900.1 | BAN16490.1 |  |  |
| QMU24799.1 | AXI82392.1 | ALE27316.1 | AAD29094.1 | QFE96906.1 | BAN16495.1 |  |  |
| QMU24807.1 | AXI82399.1 | ALE27322.1 | AAD29095.1 | QFE96912.1 | BAN16502.1 |  |  |
| QMU24815.1 | AXI82406.1 | ALE27328.1 | AAD29096.1 | QFE96918.1 | BAN16509.1 |  |  |
| QMU24823.1 | AXI82413.1 | ALE27334.1 | AAD29097.1 | QFE96924.1 | BAN16513.1 |  |  |
| QGW61694.1 | AXI82420.1 | ALE27340.1 | AAD29098.1 | QFE96930.1 | AGI16934.1 |  |  |
| QGW61700.1 | AXI82427.1 | ALE27346.1 | AAD29099.1 | QED93060.1 | AFX00029.1 |  |  |
| QGW61706.1 | AXI82434.1 | ALE27352.1 | AAD29100.1 | QED93067.1 | BAM64984.1 |  |  |
| QGW61712.1 | AXI82441.1 | ALE27358.1 | BAA35122.1 | QCW06589.1 | BAM64991.1 |  |  |
| QGW61718.1 | AXI82448.1 | ALE27364.1 | BAA33929.1 | ALL96436.1 | BAM15594.1 |  |  |
| QGW61724.1 | AXI82455.1 | ALE27370.1 | BAA33930.1 | AZO92810.1 | BAM15602.1 |  |  |
| QGW61730.1 | AXI82462.1 | AII16638.1 | BAA09953.1 | AZO92817.1 | AFG24211.1 |  |  |
| QGW61736.1 | AYV96873.1 | AIG94077.1 | BAA09960.1 | AZO92824.1 | AFC40218.1 |  |  |
| QGW61742.1 | BBG06477.1 | AIG94083.1 | BAA07026.1 | AZO92831.1 | BAK22543.1 |  |  |
| QGW61748.1 | AXF94181.1 | AHN07991.1 | BAA07028.1 | AZO92838.1 | ADY39637.1 |  |  |
| QGW61754.1 | AVV81878.1 | AFY12697.1 | BAA07029.1 | AZO92845.1 | ADY39643.1 |  |  |
| QGW61760.1 | AVW82891.1 | AFY12706.1 | BAA07030.1 | AZO92852.1 | ADN86313.1 |  |  |
| QGW61766.1 | AVO65225.1 | AFY12714.1 | CAA91370.1 | AZO92859.1 | ADI34107.1 |  |  |
| QLI46412.1 | AVA07190.1 | AEP95741.1 | P35975.1 | AYQ93078.1 | ADG96018.1 |  |  |
| QHG61094.1 | AVA07196.1 | AEP40405.1 | AAA46430.1 | AYM47332.1 | BAI60061.1 |  |  |
| QCX20599.1 | AVA07202.1 | AEP40413.1 | AAA75501.1 | ASY08097.1 | ACZ56434.1 |  |  |
| QGN67819.1 | AVA07208.1 | AEP40421.1 | AAB26147.1 | AXE15476.1 | ACE00227.1 |  |  |
| QGN67827.1 | AVA07214.1 | AEP40429.1 | P12576.1 | AXE15484.1 | ACD92998.1 |  |  |

**Supporting dataset S10.** Accession numbers of morbillivirus L sequences included in multiple sequence alignments shown in supporting figures S3 and S12.
