# Supplementary material for: Therapeutic targeting of measles virus polymerase with ERDRP-0519 suppresses all RNA synthesis activity
Source: PLoS Pathog. 2021 Feb 23;17(2):e1009371. doi: 10.1371/journal.ppat.1009371 (PMC7935272; doi:10.1371/journal.ppat.1009371)
Supplement: S11 Fig — A-F) Top scoring in silico docking poses (orange sticks) and 2-D ligand interaction projections are shown for the clinical candidate ERDRP-0519 (A-B), first generation lead AS-136 (C-D), and original screening hit 16677 (E-F). Conserved hydrogen bond interactions are predicted between all scaffolds and Y1155, N1285, and H1288. Locations of peptide 2 (purple sticks), of the intrusion (blue) and priming (red) loops, and of proposed PRNTase motifs (blue sticks) are shown. G) Stick model overlays of the docking poses of ERDRP-0519 (orange), AS-136 (red) and 16677 (blue). (PDF) [file ppat.1009371.s011.pdf]

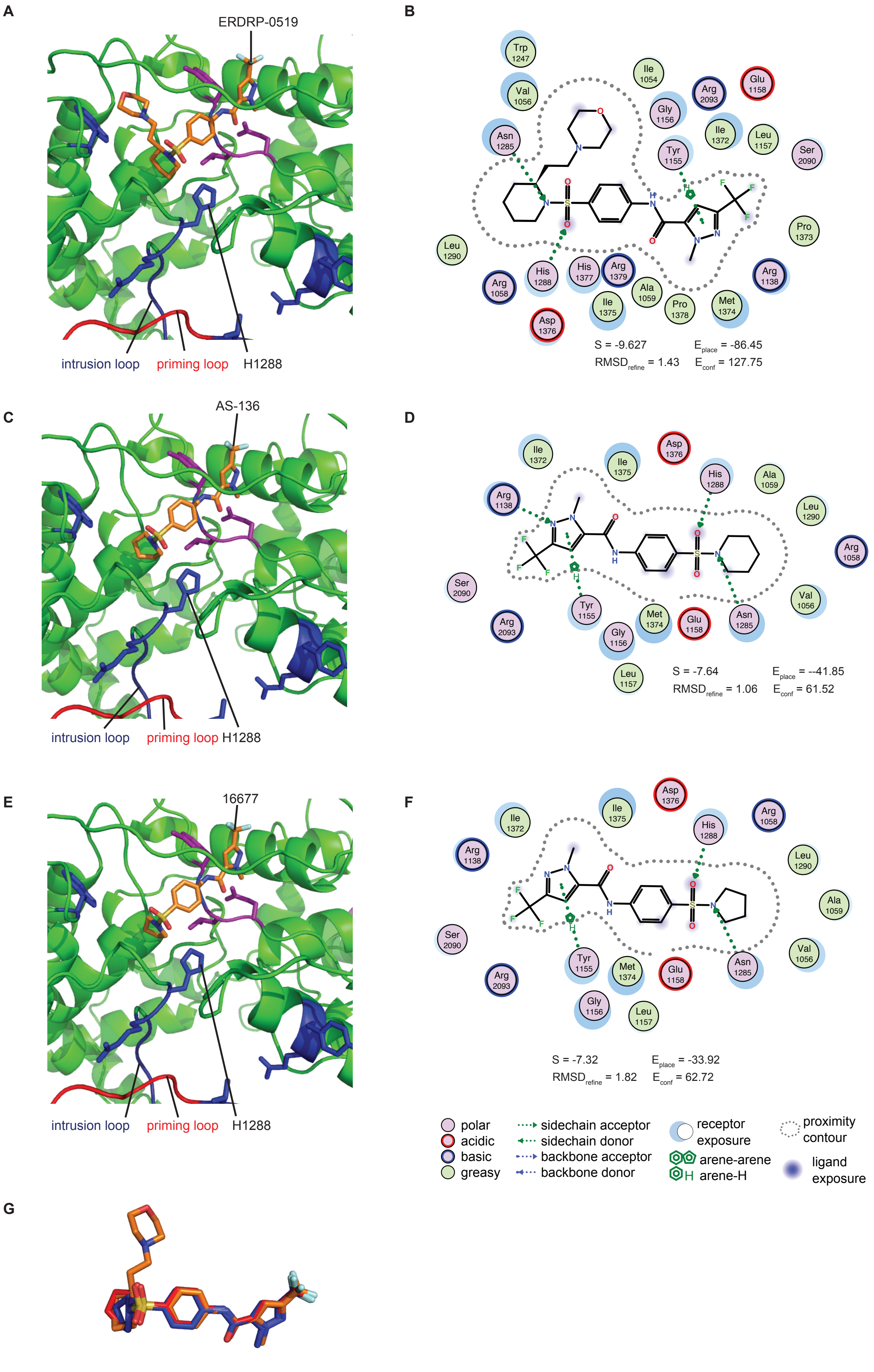

A
ERDRP-0519
intrusion loop
priming loop
H1288
B
S = -9.627
E place = -86.45
RMSD refine = 1.43
E conf = 127.75
C
AS-136
intrusion loop
priming loop
H1288
D
S = -7.64
E place = --41.85
RMSD refine = 1.06
E conf = 61.52
E
16677
intrusion loop
priming loop
H1288
F
S = -7.32
E place = -33.92
RMSD refine = 1.82
E conf = 62.72
polar
acidic
basic
greasy
sidechain acceptor
sidechain donor
backbone acceptor
backbone donor
receptor exposure
arene-arene
arene-H
proximity contour
ligand exposure
G
