# Supplementary material for: Therapeutic targeting of measles virus polymerase with ERDRP-0519 suppresses all RNA synthesis activity
Source: PLoS Pathog. 2021 Feb 23;17(2):e1009371. doi: 10.1371/journal.ppat.1009371 (PMC7935272; doi:10.1371/journal.ppat.1009371)
Supplement: Attachment — Submitted filename: Response to reviewers.docx [file ppat.1009371.s028.docx]

**Response to Reviews**

**Reviewer 1:**

*This article needs serious rewriting in its introduction. A reader that is not verse in L proteins nor Mononegavirales is unable to understand what is being attempted here. Authors have to take a great deal of care to explain the complexity of the L and its catalytic motifs A to F as well as to properly present the structural information relevant in this study. Similarly the PRNTase domain with its motifs A to E.*

We have thoroughly revised the Introduction section as recommended, now providing a succinct overview of mononegavirus polymerase domain structure and familiarizing the reader with individual catalytic motifs in the RdRP and PRNTase domains. We have furthermore highlighted the location of these motifs in revised figures 1A-C, which now better aid in illustrating the structural organization of the L protein.

Major Points

*Some aspects in the methodology needs to be clarify, controls needs to be added and some results needs to properly presented. The Materials and Methods section is at this stage incomplete. A significant part of the article and results rely on an homology model and docking procedure of which we know almost nothing about. No proper comparison between the different L protein was shown, no alignment, no sequence identity, no structural validation of the model, no docking cage information, what residues of L were involved in the docking procedure are presented. I am sorry to say I am unable to evaluate the validity of the presented in silico results. As everyone knows using in silico approach always deliver. To evaluate the degree of confidence of an in silico result the reader needs the proper methodology.*

We wholeheartedly share the reviewer’s reservations against *in silico* docking not grounded in experimental data. That is the reason why we have based our docking approach on three distinct experimental strategies, virus resistance profiling, photo-affinity labeling, and physical ligand binding to purified standard and mutant polymerase complexes. As requested, we have greatly expanded the Results and Methods sections describing the data-informed *in silico* studies. Specifically, we now show in newly added supporting figure S1 sequence alignments of MeV L with different L proteins including those used for homology model building and state sequency identify and provide validation parameters for the homology models in the newly added table 1. The expanded Methods and Results sections clarify the caging strategy applied for docking. As detailed in the following in our response to Major Points of this reviewer, we have overall expanded the Methods section of the manuscript and have added additional specificity controls to RdRP assays shown in figure 3D (newly added supporting figure S8, supporting dataset S5) as requested.

*MeV polymerase complex expression and purification procedure is done in one step. affinity chromatography. How pure is your sample ? How sure are you that you do not have contaminant ? Please provide a control gel of the purification.*

Coomassie blue-stained control gels of purified polymerase complexes are shown in figure 2A, uncut original gels are shown in the supporting dataset S1. Please note that co-purification of some cellular proteins (i.e. host cell chaperones) with viral P-L complexes is to be expected. Responding also to a related comment of reviewer 3, we have submitted our P-L preparations to mass-spectrometry analysis, which identified MeV P-L as the dominant species in the sample and provided and a specific breakdown of co-purified cellular proteins. These results are summarized in the newly generated supporting table S1 and supporting dataset S2.

*Page 9 line 153 it is said “A catalytically defective L mutant harboring an N774A substitution in the polymerase active site confirmed that RNA synthesis was MeV P-L specific and not due to co-purified cellular contaminants.” However Figure 3 C presents a gel that is badly cut but for which we can clearly see an enrich signal at 16nts specifically in the control lane. As the gel is not the raw data a doubt is present. I would like to see the uncut version of the gel. Also why the same control is not done in the case of primer extension Figure 3 D? It should be done.*

We have included uncut versions of this and all other gels generated in the study as supportive datasets, which gives the reader comprehensive access to all raw data associated with this study (supporting datasets S1-S10). With respect to the figure 3C (left panel) specifically, please note that the suspected signal at 16 nts represents bleed-through from an overly strong size marker signal – we believe that no further elaboration is required, since the uncut version of the gel shown in supporting dataset S3, page 3 clearly illustrates the point. Please also note that the same L_N774A_ control is likewise present in the other two gels shown in figure 3C (center and right panel), providing comprehensive evidence that autoradiography signals in figure 3C and associated supporting information files are RdRP activity-specific.

Unfortunately, it is known (i.e. PMID 29507309, PMID 7853486, and PMID 10729164) that RdRP complexes with comparable single point mutations in the catalytic site retain in *in vitro* polymerase assays the ability to incorporate a few nucleotides in a primer-template extension setting. Since it would therefore not have been informative to simply add this construct as additional control to the primer-extension study shown in figure 3D, we have generated for this revision an L_D773A N774A_ double mutant, expressed and purified the corresponding double-mutant P-L complexes, and tested bioactivity in the RdRP assay. As expected, this polymerase variant was unable to mediate primer elongation beyond the first bond, demonstrating P-L-specificity of autoradiography signals obtained in the primer-template extension assays (figure 3D). These additional controls are shown in the revised manuscript in the supporting figure S8 and the associated supporting dataset S5.

Minor Points

*Page 4 line 35 paramyxovirus family does not exist Paramyxoviridae*

Corrected.

*The Materials and Methods molecular biology section, is unclear I understand the procedure was already published but a brief recap of the procedure would help the reader.*

We have expanded the Molecular Biology section as requested, adding additional information that complements the references to the original publications of these plasmid systems.

*Page 11 line 214 “… part of a variable region in the viral polymerase (supporting figure S2) Figure S2 does not show that!*

Seeing this comment, we have reworded the sentence to better emphasize that supporting figure S2 (now renumbered to supporting figure S10) was intended to show the physical locations of all three peptides identified through photo-crosslinking in a structural model of MeV L, not the variable nature of the sequence stretch harboring peptide 3 across different mononegavirus L proteins.

*Figure 1B is too small and could be place in supplementary. On the other hand you need to show the catalytic sites their position relative to the different tunnels for RNA, label the motifs and keep the same reference through the whole article.*

We have enlarged figure 1B for better visibility. As detailed above, the revised figure 1C in addition shows the positions of catalytic sites and defined motifs in the RdRP and PRNTase domains. Please note that we have applied a consistent color-code of distinct polymerase domains to all structural figures and linear polymerase schematics shown in this manuscript.

**Reviewer 2:**

*The study is relevant, however, needs to address the following points.*

We thank the reviewer for this positive assessment of the importance of our work.

Major Points

*1. The mode of inhibition by ERDRP-0519 is proposed to be by blocking de novo initiation of RNA synthesis, however, the proposed binding site of ERDRP-0519 appears to be away from the priming loop. Can the authors be more specific on the structural impacts of the inhibitor binding? Also, why the impact of H589Y mutation in the proposed binding pocket is minimum? Have the authors checked the impact of H598A mutation to validate the binding pocket?*

Our study revealed four fundamental principles defining ERDRP-0519 antiviral activity: i) the compound blocks formation of the first phosphodiester bond (+1 RNA synthesis) and primer extension; ii) confirmed resistance mutations restore +1 polymerase initiation but not primer extension, identifying the former as the physiologically relevant antiviral effect; iii) resistance sites locate to conserved regions including residues in the polymerase priming and intrusion loops; and iv) photo-affinity mapping placed the ligand in direct proximity of the intrusion and priming loops, sterically blocking repositioning of the intrusion loop towards the cavity wall.

Based on these findings, we have developed the specific mechanistic hypothesis that by engaging the priming and intrusion loops the compound locks the polymerase in a pre-initiation conformation with extended intrusion loop (lines 353-360). For clarity, we have better emphasized in the revised paragraph that the extended intrusion loop is considered to both block access of template RNA to the central polymerase cavity and prevent rearrangement of the priming loop into initiation conformation.

We believe the question concerning the effect of the H589Y resistance mutation must originate from a misunderstanding. Our experiments demonstrate that the H589Y mutation has the second highest impact on mediating resistance to ERDRP-0519 of all candidates tested (Table 1), has the greatest effect on reducing compound target affinity (figure 2), and is most potent in restoring *de novo* polymerase initiation at the promoter in the RdRP assay (figure 3A,C,E). The mutation did not restore primer extension, but neither did the T776A mutation that was assessed in parallel (figure 3B,D,F). Rather than a shortcoming of the H589Y mutation, these data indicate that pharmacological interference with RNA elongation likely represents an artifact of the *in vitro* assay, using short and non-encapsidated synthetic template RNA that does not fully recapitulate physiological conditions (lines 380-392). Please note that H589Y originated from viral adaptation to this chemotype (PMID 19528268). Rather than adding engineered substitutions (i.e. H589A), we feel that concentrating on characterization of the viromics-identified mutations has greatest physiological relevance.

*2. Using single-particle cryo-em, it may be feasible to experimentally find the binding of the inhibitor to an engineered pocket in a L protein of a related virus for which the structure is already determined. If not, authors need to comment on the primary hurdle to such a study.*

For a meaningful interpretation of such a study, the L chimera with engineered binding site must be bioactive and sensitized to the inhibitor. Structural information for mononegavirus L proteins is available for rubulavirus (PIV5) L, respiratory syncytial virus L, rabies virus L, and vesicular stomatitis virus L. However, none of these L proteins is closely related to morbillivirus L, the target of ERDRP-0519, and none of these mononegaviruses with available polymerase structures is inhibited by the compound. High-affinity interaction of small-molecule ligands with protein targets typically depends on very few points of contact, requiring precise steric compatibility of the ligand and target site. Attempts to “graft” the morbillivirus L target site, which in the case of ERDRP-0519 is not formed by a single folding domain, onto a distantly related polymerase are in our experience futile, because they fail to accurately recapitulate the exact broader geometry of the central L cavity that serves as the scaffold for correct spatial positioning of the contact points.

Despite extensive efforts directed at a number of different ligands over the years, we have, in fact, succeeded only once with engineering a target site capable of supporting productive ligand binding while maintaining target protein bioactivity (PMID 32661315). However, this study sensitized an inhibitor with naturally broadened target spectrum including respiroviruses and morbilliviruses (GHP-88309) to the very closely related henipavirus L target, underscoring that the hurdles to transferring the strategy to a chemotype such as ERDRP-0519 with narrow (morbilliviruses only) and more distantly related target spectrum are major indeed, if not unsurmountable without synthetic redevelopment of the scaffold.

*3. Authors need to provide a detailed bioinformatics analysis of the pocket such as sequence and structure conservation of the region in L proteins of rhabdoviruses as supplemental material.*

Added as requested in the newly added mononegavirus L protein sequence alignment figure (supporting figure S1). As detailed in the associated figure legends, we have marked all areas containing pocket residues in the alignment through black frames.

*4. Do ERDRP-0519 and GHP-88309 inhibition exhibit synergy as their proposed binding sites and resistance mutations do not overlap?*

The reviewer is correct, these L inhibitor classes act synergistically indeed. Following this suggestion, we have experimentally explored synergy between ERDRP-0519 and GHP-88309 in a combination treatment study, systematically testing a landscape of different compound ratios centered around the respective EC_50_ concentrations. We have employed the HAS reference model (PMID 2692037) for a statistical analysis of results, which explores whether drug combination yields additional benefit beyond the effect of each compound alone. This approach identified a zone of synergistic inhibitory activity between ERDRP-0519 and GHP-88309 that was not due to heightened cytotoxicity of combination treatment (examined in parallel). These data are shown in the newly added supporting figures S4. The Results and Methods sections have been expanded accordingly.

*5. Have the authors checked the impacts of distant mutations at 1233 and 1239 positions on inhibitor binding?*

Our selection of resistance mutations for inclusion in the BLI studies was driven by the following inclusion criteria, which we believe had the highest prospect of generating an informative data set: i) a confirmed mutation of the cluster immediately surrounding the GDNQ catalytic site (represented by S768A); ii) a confirmed mutation mediating robust escape and located in proximity to the intrusion loop (represented by H589Y); and iii) a confirmed mutation not physically located within the central polymerase cavity (represented by L1170F).

Naturally, one can always test the next mutation, although considerable resources are required for P-L complex expression, purification, and experimental characterization. Without a strong hypothesis why additional mutations in loop domains (i.e. substitutions at positions 1233 or 1239) may provide new insight beyond simply phenocopying H589Y, we saw no justification to further expand the study.

Minor Points

*A summary table listing the putative pocket residues and the impact of the mutations on inhibitor binding will be helpful.*

We have expanded table 2 (formerly table 1), now showing the effect of individual resistance mutations on both polymerase activity and ligand binding as requested.

**Reviewer 3:**

*Overall, this is an interesting study containing novel results with utility for understanding both the function of MeV polymerase and the mechanism of inhibition of ERDRP-0519. The RdRp experiments are well conducted and provide important information to confirm target identification. I am somewhat less convinced by the data from the two biophysical methods (BLI and photo-affinity peptide mapping) in parts due to the low purity of the proteins and the risk of interference from contaminants and non-specific binding. I listed important control experiments and additional data needed to consolidate the biophysical results and their associated conclusions*.

We thank the reviewer for this overall appreciation of the impact of our work and excellent suggestions to further strengthen our study. As specified in detail below, we have added additional controls and/or experimental tests as suggested that we believe address the concerns associated with the BLI and photo-affinity mapping studies.

Major Points

*1) Figure 2A: What is the approximate yield and purity of the proteins? What are the multiple bands on the gel? Are these viral or non-viral proteins in Fig 2A? Please provide protein identity of the major contaminants. I am concerned about the quality of the BLI data with low purity proteins. Are there published examples of high quality BLI data obtained with impure proteins?*

As detailed in our response to major point #2 of reviewer 1, we show Coomassie blue-stained control gels of purified polymerase complexes in figure 2A in the newly added supporting dataset S1. Please note that co-purification of some cellular proteins (i.e. host cell chaperones) with viral P-L complexes is to be expected. In response to this concern, however, we have submitted our P-L preparations to mass-spectrometry analysis, which identified MeV P-L as the dominant species in the sample and provided and a specific breakdown of co-purified cellular proteins. These results are summarized in the newly generated supporting table S1 and supporting dataset S2. As additional specificity control of the BLI studies, we have tested binding of ERDRP-0519 to respiratory syncytial virus-derived P-L complexes, since respiratory syncytial virus is not inhibited by the compound. As shown in the newly added figure 2D, no binding was detected by BLI, providing additional support for the MeV P-L specificity of the BLI signals monitored in this study.

*2) Figure 2B: For the WT signal, the lowest concentration used for ERDRP-0519 is 100 nM, which is close to the reported Kd value. Can the authors repeat this experiment at lower concentration? This will help to improve the quality of the graph in Fig 2F. Also please show error bars from experimental repeats in panels F-I. In figure legend 2, please specify number of biological repeats (n) used to report the data. The authors need to make sure the wild-type BLI profile is robust and the result of multiple independent experiments because it is used for comparison with the mutant profiles and calculation of resistance level.*

Appreciating the importance of the BLI profile of the standard polymerase complex, we have tested additional ERDRP-0519 concentrations against standard MeV polymerase in the BLI assay as suggested, now spanning a study range from 3 nM to 50 μM, distributed over three biological repeats for wild type L_1708_ and two biological repeats for wild type full length L. K_D_ values showed good consistency across all five experiments with wild type L constructs, ranging from 52-140 nM. Original kinetics binding curves for the concentration tested and the associated steady-state saturation plots are shown for each of these biological repeats (figures 2B-C, supporting figure S5A-F, supporting figure S7A-B). Polymerase complexes with resistance mutations were tested in two biological repeats each. Binding curves and saturation plots are shown for each of these repeats (figures 2E-G, supporting figure S6A-F, supporting figure S7D-F). Results and/or Figure legends have been updated accordingly.

*3) Figure 5 and Line 348: I also find it surprising that escape from ERDRP-0519 inhibition with resistance mutations is mediated by secondary structural effects rather than due to primary resistance, which is unusual for non-nucleoside polymerase inhibitors. There is a risk that the photoaffinity data is misleading the identification of the binding site due to non-specific crosslinking. Therefore, in figure 5 the authors need to make sure that the peptide sequence they identified is not the result of non-specific binding and cross-linking with ERDRP-0519az. This can be done by several ways: Does ERDRP-0519az inhibit RdRp activity by P-L complex? Please show data. If ERDRP-0519az and ERDRP-0519 bind to the same site, then adding ERDRP-0519 should outcompete ERDRP-0519az cross-linking. Can you demonstrate that this is the case to indirectly indicate that the two molecules bind to the same site?*

Please note that ERDRP-0519_az_ maintained specific bioactivity against MeV L in cell-based inhibition assays (figure 5B), which we consider the gold-standard for confirming unchanged target specificity of a photo-reactive inhibitor analog. The above suggestions of the reviewer are nevertheless excellent and we have tested ERDRP-0519_az_ against P-L in the i*n vitro* RdRP assay to evaluate specificity of the photo-affinity data. We found that ERDRP-0519_az_ is bioactive in the *in vitro* RdRP assay also, blocking polymerase activity (supporting figure S9, supporting dataset S8). Since outcome of both cell-based studies and the *in vitro* RdRP were fully consistent, we did not see a need to also pursue the alternative suggestion of the reviewer to address the same question in a third way.

Minor Points

*Line 129: Why do the authors mention “mono biotinylation”? Can they provide more information on the molar ratio of protein versus EZ-Link NHS-Biotin at the labeling step? Did they measure the stoichiometry of biotin group per protein?*

We have clarified in the revised Method section that equal molar ratios were applied, resulting in statistical mono-biotinylation.

*Is the putative binding site conserved among clinical strains of MeV and other morbilliviruses? What is the level of natural polymorphism from available sequence databases? Can the authors report this information in the manuscript?*

The binding site is indeed fully conserved across MeV strains representing currently circulating genotypes and highly conserved across other morbilliviruses. This high level of sequence consistency is underlying the consistent activity of this chemotype against diverse MeV isolates and other members of the morbillivirus genus that we have reported in previous studies (i.e. PMID 19528268 and PMID 24739760). We have added a graphical representation of the degree of conservation of pocket residues based on extensive L sequence alignments representing MeV, CDV, and PPRV isolates (supporting figure S12).

*Figure 3: Does ERDRP-0519 compete with NTP and RNA binding? Does increase NTP and RNA concentrations change IC50 value, as predicted by docking pose of ERDRP-0519 in the template RNA channel? (line 368).*

This suggestion is very interesting, but unfortunately execution of the experiment in the *in vitro* RdRP assay is not feasible. *In vitro* assay conditions including NTP and RNA concentrations have been carefully optimized for maximal signal, and changing any parameters drastically affects polymerase bioactivity (and hence intensity of the product signals). Altering relative NTP or RNA ratios would either result in complete loss of signal or, at the minimum, require recalibration of the assay for every new condition tested, which would undermine the direct comparison the reviewer seeks.

Whereas we therefore cannot follow this suggestion, please note that experimental conditions used in *de novo* transcription (figure 2C) and primer-template extension (figure 2D) assays are drastically different from each other and from physiological concentrations. Despite this wide range of assay conditions, inhibitory concentrations of ERDRP-0519 were remarkably consistent across both types of *in vitro* (figures 2E and 2F) and the *in cellula* (Table 2) assays, suggesting that compound and substrate binding are not competitive within this concentration range.

**Editorial Instructions**

Figure Files:

*While revising your submission, please upload your figure files to the Preflight Analysis and Conversion Engine (PACE) digital diagnostic tool, https://pacev2.apexcovantage.com.*

Figures have been subjected to the PACE tool as requested. All main figures submitted have been PACE validated.

Data Requirements:

*Please note that, as a condition of publication, PLOS' data policy requires that you make available all data used to draw the conclusions outlined in your manuscript. Data must be deposited in an appropriate repository, included within the body of the manuscript, or uploaded as supporting information. This includes all numerical values that were used to generate graphs, histograms etc.*

We have generated and included a multi-tab source data file (supporting dataset S9) that contains all numerical raw data (organized by figure #). Uncropped copies of all gels shown in the manuscript are provided in supporting dataset S1 and supporting datasets S3-S8.

Reproducibility:

*To enhance the reproducibility of your results, PLOS recommends that you deposit laboratory protocols in protocols.io, where a protocol can be assigned its own identifier (DOI) such that it can be cited independently in the future. For instructions see http://journals.plos.org/plospathogens/s/submission-guidelines#loc-materials-and-methods*

All experimental protocols are described in the expanded, revised Methods section.
